# Diversity and Novelty of Venom Peptides in Vermivorous Cone Snails, Subgenus *Rhizoconus* (Gastropoda: Mollusca)

**DOI:** 10.3390/md23070266

**Published:** 2025-06-26

**Authors:** Christine Marie C. Florece, Quentin Kaas, Neda Barghi, Arturo O. Lluisma

**Affiliations:** 1 Marine Science Institute, University of the Philippines, Quezon City 1101, Philippines; ccflorece@up.edu.ph; 2 Philippine Genome Center Visayas, University of the Philippines Visayas, Iloilo 5023, Philippines; 3 Syngenta Crop Protection AG, 4332 Stein, Switzerland; quentin.kaas@syngenta.com; 4 Max Planck Institute for Evolutionary Biology, 24306 Plön, Germany; barghi@evolbio.mpg.de

**Keywords:** cone snails, conopeptides, worm-hunting, transcriptomics, venom gland

## Abstract

A large majority of cone snails (a species in the genus *Conus*) are vermivorous (worm-hunting), but the diversity and bioactivity of their venom peptides remain largely unexplored. In this study, we report the first venom gland transcriptomes from two species in the *Rhizoconus* clade, *Conus capitaneus* and *Conus mustelinus*, and a new *Conus miles* transcriptome from a specimen collected in the Philippines. From the set of assembled sequences, a total of 225 *C. capitaneus*, 121 *C. miles*, and 168 *C. mustelinus* putative peptide toxin transcripts were identified, which were assigned to 27 canonical gene superfamilies in *C. capitaneus* and 24 in *C. miles* and in *C. mustelinus*. Most of these venom peptides are novel, and some exhibit new cysteine patterns. Clustering also revealed 12 putative novel gene superfamilies, highlighting the diversity of uncharacterized venom peptides in this group. The O1-, M-, O2-, and con-ikot-ikot superfamilies were the most abundant, while gene superfamilies such as D and G2 were highly expressed. Several hormone-like conopeptides were also identified in this study, revealing the vast diversity of conopeptides from the *Rhizoconus* species.

## 1. Introduction

Cone snail species (genus *Conus*) use diverse predatory strategies underpinned by potent and rapidly evolving cocktails of peptide toxins [1]. Worm-hunting cone snails constitute the largest group, representing 72% of all the known species in this genus [2]. The evolution of the two other major groups, i.e., fish- and mollusk-hunting species, from the ancestral worm-hunting cone snails during the Miocene period marked a pivotal point in species diversification in this genus [3,4,5]. The diet of most worm-hunting cone snails typically consists of eunicid, terebellid, and capitellid polychaetes [6,7]. In contrast, species within the *Rhizoconus* clade are reported to prey on fireworms or amphinomic polychaetes, suggesting that they use different sets of toxins, at least for predation [7]. So far, the venom gland transcriptomes of only three *Rhizoconus* species, namely *C. miles*, *C. rattus*, and *C. vexillum*, have been analyzed, highlighting a significant knowledge gap in this area.

The peptides found in cone snail venoms are called conopeptides, and conotoxins are a specialized subset of conopeptides characterized by the presence of more than one disulfide bond [8]. Conopeptides, which are ribosomal peptides, are expressed as precursors that comprise three regions: (i) an N-terminal signal sequence, which is highly conserved within gene superfamilies; (ii) propeptide regions, which are essential for folding and/or maturation; and (iii) a hypervariable toxin region, which is excised from the precursor during maturation and forms the bioactive peptide [9,10,11]. Investigating the repertoire of conopeptides from lesser-studied clades could result in the discovery of not only biological and biochemical insights, such as novel cysteine frameworks (folds) and new gene superfamilies, but also new potential biomedical applications for these peptides [12,13].

Transcriptomics has significantly advanced the study of venom and toxin evolution in venomous species [14], such as cone snails [15,16]. Compared to proteomics or Sanger sequencing, massively parallel sequencing technologies achieve higher depth and coverage, and transcripts with low expression levels can be identified [17], enabling a high resolution of conopeptide diversity in cone snails [18,19].

This study aims to expand our understanding of conopeptide diversity by performing transcriptome assembly analysis to characterize the venom peptide components of three worm-hunting species of *Rhizoconus*: *C. capitaneus*, *C. miles*, and *C. mustelinus*.

## 2. Results

### 2.1. Pre-Processing, Assembly, and Annotation Metrics

Raw sequencing reads of 64 M bp for *C. capitaneus*, 56 M bp for *C. miles*, and 83 M bp for *C. mustelinus* were subjected to k-mer correction using rcorrector [20]. Subsequently, these corrected reads were filtered and trimmed using AfterQC [21] with a minimum quality score of 20. After pre-processing, 98.3%, 99.7%, and 98.8% of the high-quality reads were retained for *C. capitaneus*, *C. miles*, and *C. mustelinus*, respectively. Clean reads were assembled using Trinity v2.11.0 [22]; assembly statistics are shown in Table 1.

**Figure 1:**
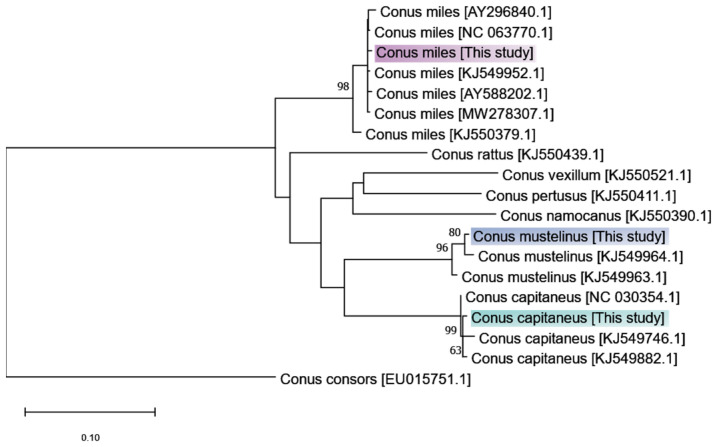
Reconstructed maximum likelihood phylogeny based on mined and assembled cox1 fragments from the raw reads of the three species analyzed in this work, together with sequences from related *Rhizoconus* species retrieved from GenBank. The maximum likelihood tree was generated using the HKY + G + I substitution model. Node values represent bootstrap support values (1000 replicates).

A maximum likelihood method was used to reconstruct a phylogenetic tree using COI fragments mined and assembled from the raw reads of *C. capitaneus*, *C. miles*, and *C. mustelinus* together with CO1 sequences of other vermivorous cone snails obtained from NCBI (Figure 1). The phylogenetic tree shows that the CO1 sequences recovered from the transcriptome data unambiguously grouped with sequences from the target species, confirming the taxonomic identity of the specimens.

### 2.2. Summary of Identified Conopeptides

This study reports for the first time the de novo transcriptome assemblies of the venom ducts of *C. capitaneus* and *C. mustelinus*, as well as an improved assembly of *C. miles*. Totals of 225, 121, and 168 putative conopeptide precursor sequences were identified for *C. capitaneus*, *C. miles*, and *C. mustelinus*, respectively. Most of these sequences are reported for the first time (i.e., no identical sequences were found in the NCBI Nr, UniProt/SwissProt, and ConoServer databases), and some possess new cysteine patterns. We then summarized and named these predicted conotoxins from the three *Rhizoconus* species as Cpt001-225, Mil001-121, and Mus001-168 (Supplementary File S1).

### 2.3. Conopeptide Diversity

**Figure 2:**
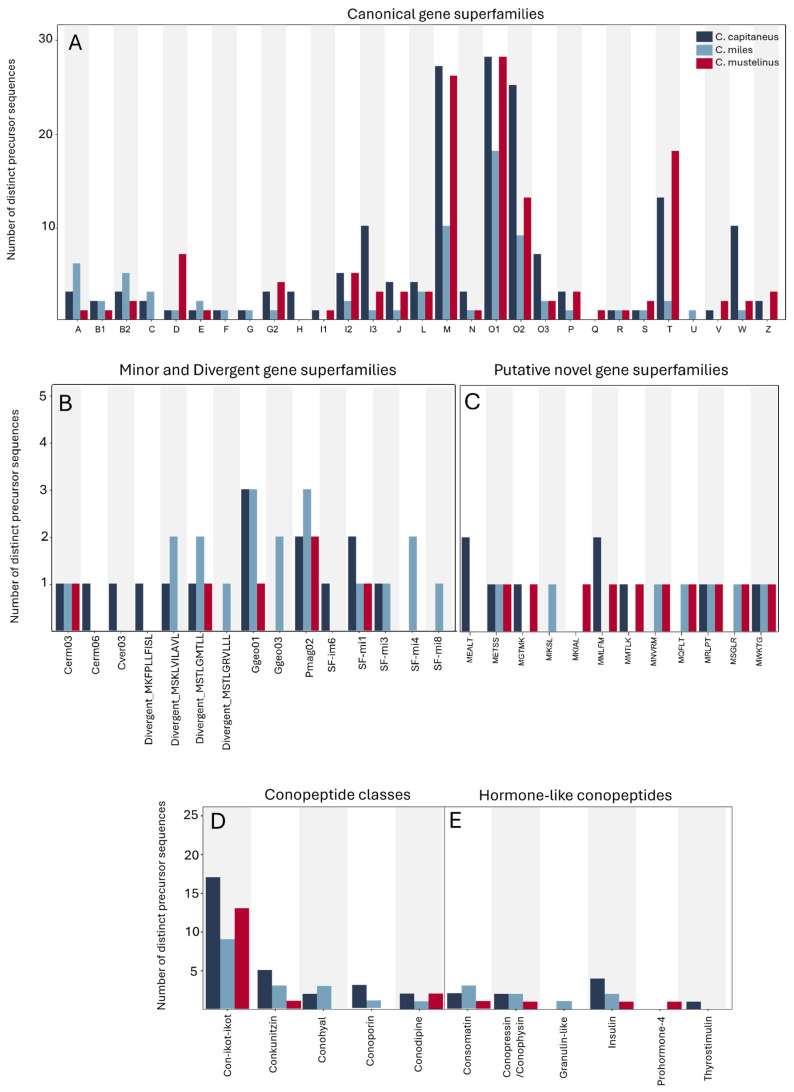
Frequency of conopeptide superfamilies identified in the venom gland transcriptomes of *C. capitaneus*, *C. miles*, and *C. mustelinus*. (**A**) Canonical conotoxins; (**B**) minor gene superfamilies; (**C**) putative novel gene superfamilies; (**D**) conopeptide classes; (**E**) hormone-like conopeptides.

Values for various diversity metrics computed for the predicted venom peptides from each species are shown in Table 2, indicating high diversity and a relatively uniform distribution. A summary of the identified gene superfamilies is shown in Figure 2; 29 canonical gene superfamilies and minor and divergent gene superfamilies were identified. This study also identified several non-paralytic conotoxins, including members of the con-ikot-ikot and conkunitzin classes, as well as conoporin (pore-forming conopeptide that disrupts cellular integrity), conodipine (phospholipase A2 enzyme), conohyal (targets extracellular matrix, aiding in the spread of venom), contulakin (neurotensin receptor agonist), and several hormone-like conopeptides (Figure 2). Moreover, 12 unclassified groups tagged as putative novel gene superfamilies due to their unique signal peptide sequences, whose sequence similarity to the other groups was below the universal identity threshold of 75%, were also observed.

**Figure 3:**
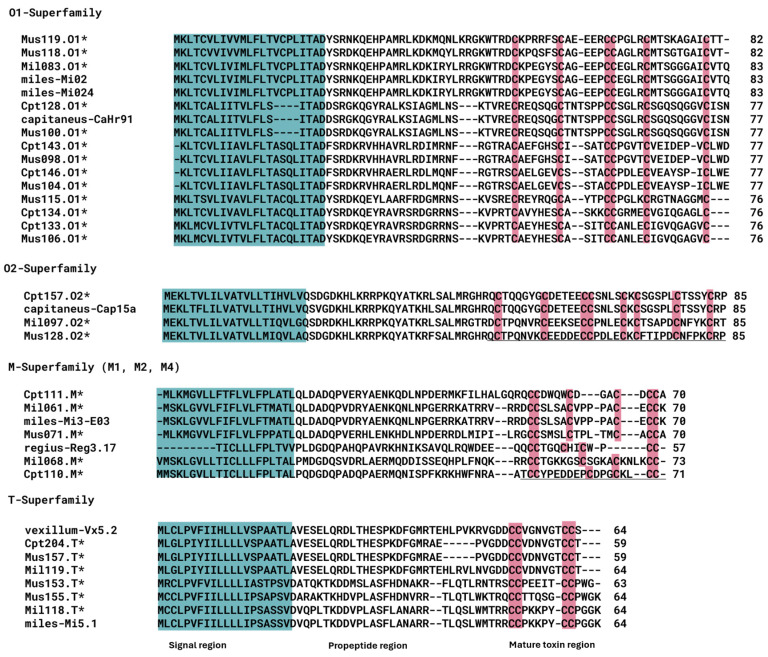
Representative members of the O1-, O2-, M-, and T-superfamilies. The signal peptide sequence is highlighted in blue, the toxin region is underlined, and cysteine residues are emphasized in red. Asterisk (*) represents sequences identified in this study.

For all three *Rhizoconus* species analyzed in this study, the O1-, M-, T-, and O2-gene superfamilies were the most abundant, and there was a notable presence of cysteine-rich con-ikot-ikot peptides (Figure 2). Most members of the O1- and O2-superfamilies exhibit the conventional VI/VII (C-C-CC-C-C) cysteine framework, which forms a stable three-disulfide inhibitor cystine knot (ICK) motif that is crucial for peptide stability and function (Figure 3) [23].

In addition to the VI/VII framework, other cysteine frameworks were also observed in O2-superfamily members, including type XV (C-C-CC-C-C-C-C), type XVI (C-C-CC), type XII (C-C-C-C-CC-C-C), and a single disulfide bond (C-C) commonly found in O2/Contryphans (Supplementary File S2). Among the T-superfamily peptides, the classical type V (CC-CC) framework was observed, as well as an unusual ten-residue cysteine arrangement (CC-CC-C-C-C-C-C-C), which has not been widely reported. While diverse in its cysteine patterns, the M-superfamily was primarily characterized by the type III (CC-C-C-CC) framework. The type III M-superfamily members were further classified into subgroups M1, M2, and M4 (Figure 3) based on the varying number of residues between the fourth and fifth cysteine residues [24].

Despite only a few previously identified precursor sequences, the W-superfamily stood out as one of the most diverse gene superfamilies in this study, particularly in *C. capitaneus*. Other abundant gene superfamilies included I (subfamilies I1, I2, and I3), D, L, and O3, all well-represented across the three species analyzed. In addition to these major superfamilies, numerous conotoxins belonging to the A-, B2-, C-, E-, F-, G2-, J-, P-, R-, S-, Y-, and Z-superfamilies were also predicted in the venom gland transcriptomes of the three species.

### 2.4. Relative Gene Expression Profile of Rhizoconus

In this study, conopeptide expression accounted for approximately 19% of all the transcripts in the *C. capitaneus* venom gland transcriptome and higher in the transcriptomes of *C. miles* and *C. mustelinus* (56% and 51% of the total TPM, respectively), as detailed in Table 3. The total expression level was calculated by summing the TPM values for all the conopeptides over the sum of the total TPM of the assembly [25], and the relative expression level was determined by normalizing the total superfamily expression with the aggregated TPMs of all the conopeptides.

**Figure 4:**
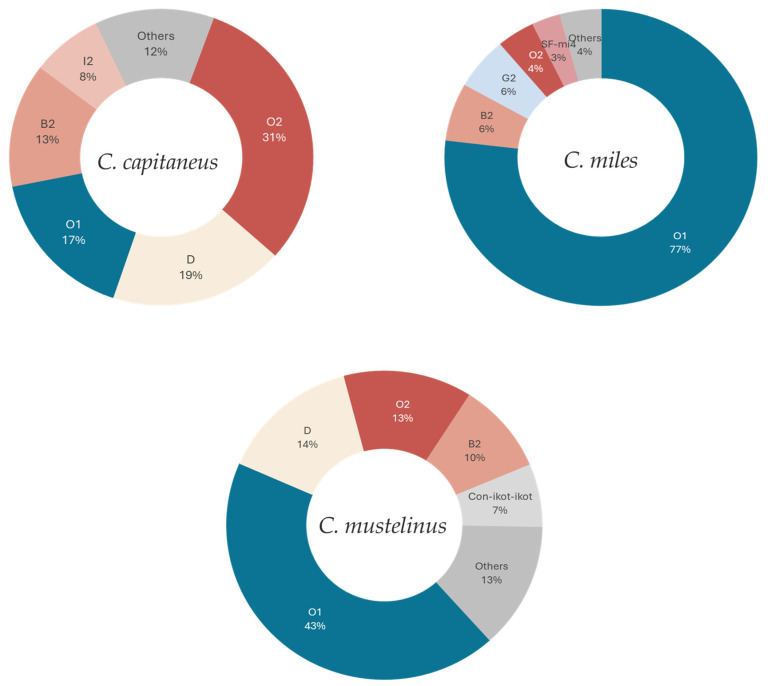
Comparison of relative gene expression levels of the most highly expressed conopeptide gene superfamilies in *C. capitaneus*, *C. miles*, and *C. mustelinus*. The conopeptide gene superfamilies classified as “Others” are detailed in Supplementary File S4.

In a comparative analysis focusing on the gene superfamily expression across species, three superfamilies, O1, O2, and B2, showed high expression levels in all the examined species (Figure 4). The O1- and O2-superfamilies in the three *Rhizoconus* species also have the greatest number of distinct conopeptide sequences as well as the most highly expressed gene superfamilies, suggesting that toxins that are highly expressed in the transcriptome are typically the first to be biochemically characterized due to their abundance in the venom [27]. However, a different case was observed in other gene superfamilies with less than ten members (Supplementary File S5). For example, in *C. capitaneus*, the D-superfamily with only one member had surprisingly the second highest expression level (19%), while the I2-superfamily with only five members contributed 8% to the expression profile. With only one transcript, the G2-superfamily (TPM 32,050.46, Table 4) from *C. miles* accounted for 6% of the expression (Supplementary File S4). In addition, the SF-mi4 superfamily has a high expression level with just two conotoxins accounted for. This pattern of low abundance but high expression was also evident in the B2- and D-superfamilies in *C. mustelinus*. Other notable findings include several identified hormone-like conopeptides of low expression levels (Table 4).

### 2.5. Gene Superfamilies with Sequence Similarity to Other Previously Studied Rhizoconus Conotoxins

**Figure 5:**
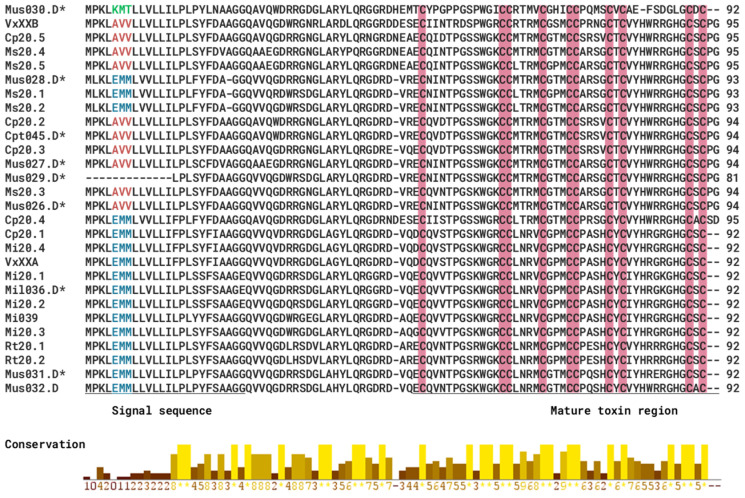
Multiple sequence alignment of D-superfamily conotoxins. The signal and mature peptide regions are underlined, and cysteine residues are highlighted in pink. Asterisk (*) represents the sequences identified in this study. Colored amino acids indicate a signal peptide motif.

**Figure 6:**
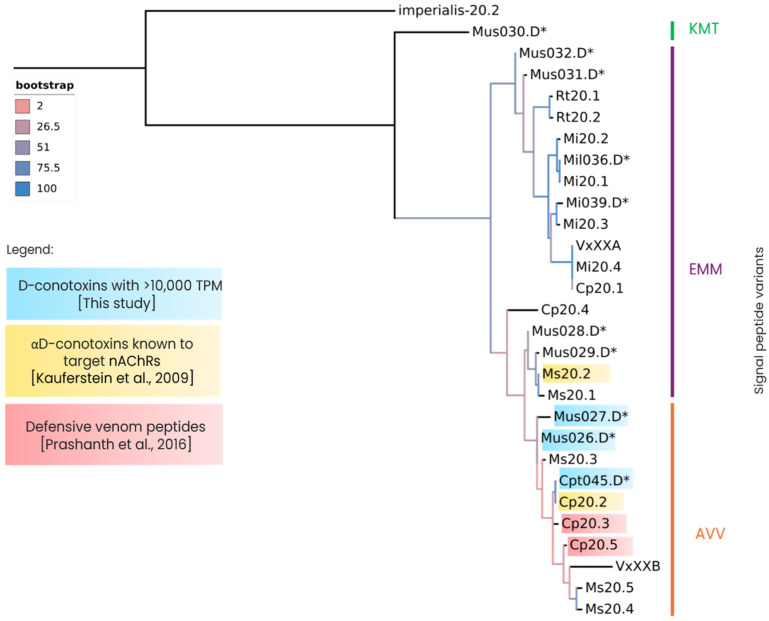
Phylogenetic analysis of D-superfamily conotoxins and previously identified D-conotoxins [28,30]. The amino acid sequences were aligned, and a maximum likelihood tree was generated using the Dayhoff substitution model and a bootstrap value of 1000. Asterisk (*) represents the sequences identified in this study.

Conopeptides that act as antagonists to neuronal nAChRs are classified as α-conotoxins, most of which belong to the A-, M-, S-, C-, and D-superfamilies [28]. Figure 5 shows several αD-conotoxin-like sequences with high sequence similarity to previously reported conotoxins, along with three identified signal peptide motifs: EMM, AVV, and a putative novel motif, KMT (Figure 6). Notably, the highly expressed Cpt045.D (TPM 35273.39) and Mil036.D (TPM 2053.48) exhibit 100% sequence identity with previously characterized *C. capitaneus* conotoxin Cp20.2 and *C. miles* conotoxin Mi20.1 [29], respectively (Figure 5; Figure 6). For *C. mustelinus*, conotoxins Mus028.D and Mus029.D share over 95% identity with the previously identified Ms20.2 [28], while Mus026.D (TPM 53931.68) and Mus027.D (TPM 14774.67) share 89.2% and 83.9% identity, respectively [29].

Aside from the D-superfamily, some interesting sequence similarities were observed for the O1- and O2- superfamilies. In particular, the highly expressed Cpt128.O1 (TPM 30796.89) is identical to the previously identified CaHr91 precursor [31]. Similarly, conotoxins Cpt156.O2 (TPM 24241.42) and Cpt157.O2 (TPM 11814.29) share 92% and 97% identity with another previously identified Cap15a (C8CK77) from the O2-superfamily (Figure 3).

**Figure 7:**
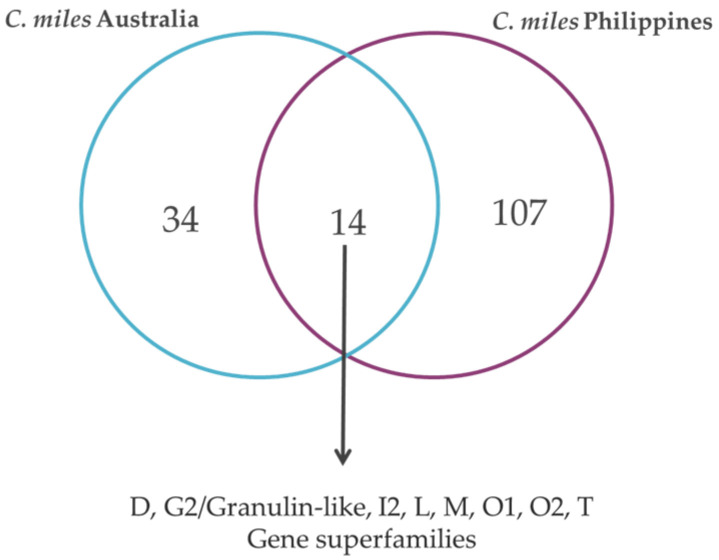
Venn diagram representation of conotoxin precursor sequences (identified via transcriptomics) that were shared between or unique to the two *C. miles* individuals from Australia and the Philippines. For the shared sequences, the different gene superfamilies are shown.

In this study, we also report thirty-seven precursors identified in *C. miles* with sequence similarity to the validated sequences in the previously reported *C. miles* venom gland transcriptome of a specimen from the Great Barrier Reef, Australia [19]. Of these, fourteen are identical (Figure 7), nine sequences have 82–99%, and fourteen sequences have <70% sequence similarity (Supplementary File S3).

On the other hand, 12 conotoxin sequences in the *C. miles* transcriptome were identical in sequence to the mature regions of previously reported peptides in the *C. miles* proteome [19], as shown in Table 5.

In addition to the venom gland transcriptome of *C. miles* from Australia, the same sequences, Mil078.O1 and Mil082.O1, were found to be identical to the mature toxin region of the previously identified MiEr95 sequence identified using cDNA cloning and sequencing from *C. miles* isolated from the South China Sea of Hainan Province [31] (Supplementary File S3). Furthermore, sequence comparisons reveal that Mil084.O1 shares 98.6% identity with the MiEr93 precursor [31], while 12 precursor sequences have <85% similarity. It was also observed that *C. miles* Mil001.A (TPM 9) and Mil002.A (TPM 5.56) have sequence similarities to the previously identified A-conotoxins Sm1.3 and Sm1.1, respectively, from fish-hunting *C. stercusmuscarum* [1].

**Figure 8:**
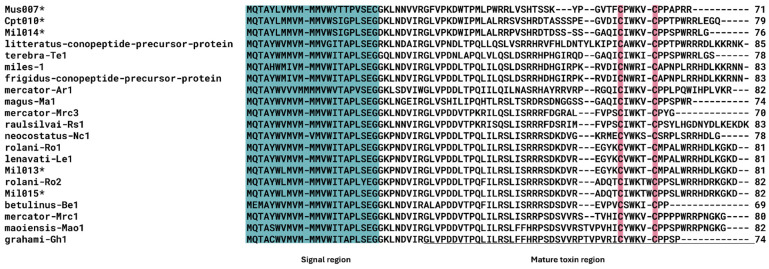
Multiple sequence alignment of the putative consomatin conopeptide sequences. The signal peptide sequence is highlighted in blue, the toxin region is underlined, and cysteine residues are emphasized in red. Asterisk (*) represents sequences identified in this study.

Notably, several hormone-like conopeptides, such as *C. miles* Mil013 and Mil015, were identified as putative consomatin peptides, a cysteine-poor conopeptide in the C-superfamily, as the first representatives of this superfamily in *Rhizoconus* (Figure 8). Mil013 and Mil015 conopeptide precursors have identical mature peptide regions to consomatin-Ro1 and Ro2, respectively [32]. The only difference in these precursor sequences from *C. miles* and *C. rolani* is in the signal peptide sequence: two and one positions differ for Mil013/Ro1 and Mil015/Ro2, respectively (Figure 8). These two putative consomatins identified in *C. miles* have transcripts per million (TPM) values below 10, suggesting a low transcription level (Table 4).

**Figure 9:**
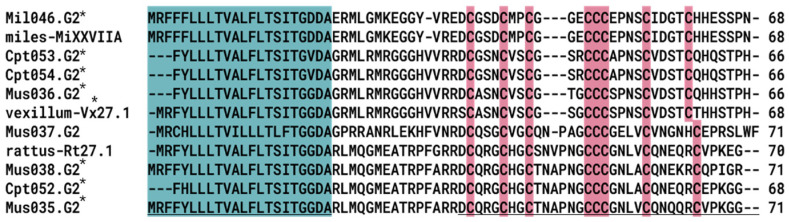
Multiple sequence alignments of granulin-like conopeptide from *C. miles*. The signal peptide sequence is highlighted in blue, the toxin region is underlined, and cysteine residues are emphasized in red. Asterisk (*) represents sequences identified in this study.

The granulin-like ϕ-MiXXVIIA was first discovered in *C. miles* [33] and was also identified in this study (Figure 9). This ϕ-MiXXVIIA has eight cysteine residues (C-C-C-CCC-C-C), including a rare vicinal cysteine residue triplet (CCC) (Figure 9).

## 3. Discussion

Advances in high-throughput transcriptome sequencing and bioinformatics have significantly accelerated the discovery of conotoxins, essential for understanding the diversity of venom components in cone snails and developing novel pharmacological tools [24]. This study exposes the diversity of conopeptides in the venom of three vermivorous species in the *Rhizoconus* clade via analysis of the first venom duct transcriptomes from *C. capitaneus* and *C. mustelinus* and an additional venom duct transcriptome from *C. miles*.

Numerous putative conopeptide precursor sequences were identified for these species: 225 *C. capitaneus*, 121 *C. miles*, and 168 *C. mustelinus*. Of the 67 distinct superfamilies identified from the three transcriptomes, 33 were shared by all three species, 16 by two, and 18 were found in only a single species. In addition, this study reports putative novel conopeptides as well as conopeptides first reported in the *Rhizoconus* clade. The power of sequencing technologies and advanced algorithms has facilitated the identification of novel sequences, gene superfamilies, and cysteine frameworks, as evidenced by the 12 putative novel gene superfamilies identified in the study. The ConoServer database records, as of January 2025, list only twenty-eight protein precursors and twenty-three nucleic acid sequences for *C. capitaneus*, seventy-five protein precursors and sixty-four nucleic acid sequences for *C. miles*, and seven protein precursors and two nucleic acid sequences for *C. mustelinus*. Thus, the findings of this study substantially expand the conopeptide libraries in these worm-hunting species.

The diversity of the identified conopeptides in each *Rhizoconus* specimen analyzed in this study is high, as seen in the number of gene superfamilies, providing new insights into the potential predatory behavior and ecological strategies of these worm-hunting cone snails. The diversity and composition of conopeptides in each *Conus* species are closely linked to their prey preferences; worm-hunting cone snail species that coexist in the habitat have a distinct diet, preferentially targeting specific polychaete worms [6,34]. This dietary specialization suggests that each species has evolved unique venom profiles tailored to capture different prey groups [6,35]. For example, it was previously observed that *Rhizoconus* species predominantly prey on fireworms or amphinomic polychaetes [7]. This specialization likely influences the venom composition of these snails, leading to a more specific array of conopeptides than species with a broader prey range [36,37].

Superfamilies O1, M, T, and O2 represent the most abundant gene superfamilies in the venom of *C. capitaneus*, *C. miles*, and *C. mustelinus*. These superfamilies have been previously reported to constitute cone snails’ basic venom toolkit for predation and defense [17,38]. In addition, the diversity of the O1-superfamily, its structural characteristics, and its selective targeting of various ion channels [39] are thought to play important roles in the evolutionary success of the family Conidae [27].

We found no correlation between the number of transcripts in a gene superfamily and the gene expression level. For example, superfamilies O1 and O2 are highly expressed, but these superfamilies also comprise a relatively large number of members; i.e., they have significant peptide diversity. In contrast, the D-superfamily is highly expressed but only has a few representatives. In a previous study of *Rhizoconus* species *C. rattus*, the L-superfamily was found to have the most members, but one of the conotoxins in this superfamily also had the highest expression (Rt_L_3) [25]. Therefore, *C. capitaneus* in this study differs from the other two *Rhizoconus* species because the most highly expressed putative toxin does not belong to the most populated gene superfamily in terms of a number of different transcripts. This absence of correlation was also suggested in other *Conus* species like *C. ebraeus*, *C. marmoreus*, *C. sponsalis*, and *C. virgo* [25]. Although expression profiles were obtained from a single specimen per species, the observed differences between species are preliminary and require further validation. This type of analysis is important for investigating the emergence of new toxins by gene duplication; however, such inquiries are beyond the scope of this study.

In addition to the previously mentioned gene superfamilies and conopeptide classes, we identified a diverse array of hormone-like conopeptides. These conopeptides have low expression values; i.e., they are not abundant venom components. Nonetheless, these peptides could still play important roles in envenomation [40].

The discovery of non-paralytic conotoxins in this study, including con-ikot-ikots, suggests a broader functional diversity, potentially involving complex prey capture strategies beyond direct paralysis [41,42]. These toxins may play a role in complex prey capture strategies as they are known to modulate AMPA receptors [43]. Additionally, con-ikot-ikot has been identified in other worm-hunting species, such as *C.* (*Elisaconus*) *litteratus* [16], *C.* (*Splinoconus*) *lenavati* and *C.* (*Splinoconus*) *tribblei* [44], and *C.* (*Lividoconus*) *quercinus* [45].

Several new αD-conotoxin-like sequences were identified, which is interesting because these toxins have unique structures and modes of action on nAChRs [28,46,47,48,49]. This is important because nAChRs are potential targets for treating various diseases and conditions, such as epilepsy, Alzheimer’s, and Parkinson’s [50,51]. Moreover, αD-conotoxins have hitherto only been found in vermivorous species, and the high expression levels of certain αD-conotoxins (Cpt045 and Mus026 in this study) suggest that they are functionally important. In contrast to α-conotoxins, which are 10–30 amino acid monomeric toxins and approximately 1–4 kDa [52], αD-conotoxins are pseudo-homodimers and much larger, with each chain having 47–50 residues and a mass of approximately 11 kDa [28]. These peptides are known for their distinctive disulfide connectivity and structural complexity, which allow them to form highly stable and rigid conformations, potentially enhancing their resistance to enzymatic degradation and prolonging their activity [43]. Previous studies reported αD-conotoxins target nAChRs in a distinct manner from other conotoxins, and their binding site only partially overlaps that of α-conotoxins [46]. The selective interaction with nAChRs of αD-conotoxins may be involved in immobilizing worm prey by disrupting neural communication, thereby providing an efficient means of prey capture or defense [53]. Given the αD-conotoxins activity on the α7 nAChR subtype in mammalian assays and their expression in the proximal section of the venom gland [30], they could, therefore, also be involved in a defensive strategy, potentially deterring predators and competitors, such as fish, which may attempt to exploit these snails’ foraging grounds [54].

The absence of A-superfamily toxins in several worm-hunting cone snail species [55,56] implies that this superfamily may not be vital for vermivory [15]. By contrast, this study identified nine putative conopeptide precursors belonging to the A-superfamily, all of which have low expression levels. It remains uncertain whether all individuals of these species exclusively express the conotoxins identified in this study. Notably, a previously reported A-superfamily conotoxin, Mi1.1, has been identified in *C. miles* [57]. Interestingly, the putative A-superfamily conotoxins detected in *C. miles* in this study share an identical mature peptide region with those found in *C. stercusmuscarum*, a fish-hunting species from the *Pionoconus* subgenus [1]. This similarity is unexpected given the phylogenetic distance between *Pionoconus* and *Rhizoconus* [58]. Since this study sequenced only one individual, further validation of the identified A-superfamily conotoxins is necessary. To date, several A-superfamily conopeptides have been discovered in worm-hunting cone snails: *C. arenatus* [59], *C. caracteristicus* [45], *C. coronatus* [60], and *C. regius* [61].

The presence of these conopeptides confirms the previous report [62], which predicts the presence of the A-superfamily in all *Conus* species [19,25,63]. Our study also identified S-Superfamily conotoxins in the *Rhizoconus* sub-genera for the first time. The presence of these gene superfamilies is particularly interesting because most α-conotoxins that act as antagonists to neuronal nAChRs belong to these gene superfamilies and are considered to be pharmacologically important [28,51].

An interesting finding is the presence of cysteine-poor conopeptides, specifically consomatins of the C-superfamily, observed in a *Rhizoconus* species. *C. miles* sequences show sequence similarity to consomatin-Ro1 and Ro2 isolated from *C. rolani* [32]. Minor differences in amino acids were observed between these sequences. The amino acid substitutions observed in *C. miles* appear to result from single-nucleotide substitution at heterozygous sites and, depending on which nucleotide is found at the SNP position, would result in precursor peptides that are either identical in sequence to its homologs in *C. rolani* or different by only one residue. Supporting mRNA reads provide evidence of these substitutions. A previous study conducted genome and transcriptome mining of 19 worm-hunting cone snail species [64], identifying consomatins in *C. miles* and 10 other worm-hunting clades, hinting that consomatins expressed in some cone snail species may be involved in an SSRP-like signaling system in annelids. It was hypothesized that consomatins are part of an “ambush-and-assess” predation strategy, where the toxins act slowly [32]. Additionally, consomatin Ro1, the first vertebrate hormone somatostatin (SS) analog, is derived from the venom of *C. rolani* **[32]**. Consomatin Ro1 has similar conformations to that of Octreotide, a pharmacological SS analog, which displays D-Trp at analogous positions to Ro1. Somatostatin analogs, such as consomatins, attract interest because they have the potential to be used for the treatment of pain, cancer, and endocrine disorders [32]. Since only one specimen for each species was sequenced and analyzed in this study, further research is needed to confirm these results. Nonetheless, the findings have significant implications, from the similarity of the biochemical function of specific venom components to the overlap of predation capabilities.

Several granulin-like conopeptides of the G2 superfamily were also observed in the transcriptomes. Granulins are growth factor proteins involved in cell growth, repair, and wound healing [65]. Previous studies in *C. miles* [33] demonstrated anti-apoptotic activity in the granulin-like ϕ-MiXXVIIA conotoxin. The identified granulin-like conopeptides in this study could also potentially exhibit anti-apoptotic properties similar to those observed in ϕ-MiXXVIIA conotoxin, warranting further functional characterization to explore their potential pharmacological significance.

The conopeptide sequences identified in this study in the *C. miles* transcriptome significantly expanded the known diversity of conopeptides in this species based on a previous analysis of transcriptome (cDNA sequencing using 454 pyrosequencing) and proteome data obtained from a *C. miles* specimen collected in the Great Barrier Reef, Australia [15], as well as the five O1-sequences obtained from cDNA cloning and sequencing from *C. miles* specimens collected from Hainan Province [31]. The venom gland transcriptome from the *C. miles* Australia study [15] reported eight gene superfamilies (O1, O2, D, M, T, I2, L, and P) and eight putative new gene superfamilies (SF mi1–mi8). Here, we report 24 canonical gene superfamilies and other hormone-like conopeptides, further diversifying the conopeptide profile of the *C. miles* venom gland. A comparison of the two venom gland transcriptomes revealed that 18% of the predicted conopeptides in this study showed 97–100% sequence similarity to those identified in the previous study, and approximately 13% were identical in their mature (toxin) regions. This cross-validation not only confirms that the sequences predicted in this study are indeed present in the venom of *C. miles* [15] but also showcases the novel conopeptide sequences from *C. miles*. Furthermore, this study demonstrates a potential link between distinct geographical populations of *C. miles*—from the Great Barrier Reef, Australia, and the Philippines—and, while the findings suggest that *C. miles* from different habitats may exhibit variations in their venom profiles, further studies are needed to validate these observations. Differences in the conopeptide profiles have also been reported in several *Conus* species such as *C.* (*Chelyconus*) *purpurascens*, where specimens collected from diverse locations—including Clipperton Island [66], the Pacific shores of Costa Rica [67], Panama [68], and Ecuador [69]—displayed distinct venom profiles. The study of *C. purpurascens* also highlighted distinct venom components in individuals from different regions, suggesting an influence of geographical locations in shaping conopeptide diversity [70]. The venom profile of cone snails is known to be shaped by several factors, such as the developmental stage [71], habitat and geographical location [72,73], diet [25], and threats [74], but also by the state of the specimen during capture and the methods of sample preparation [75,76]. The similarities and differences may be attributed to the well-documented intraspecific variation observed in cone snails [77,78,79]. It is, however, important to note that the venom gland transcriptomes of *C. miles* analyzed in this study and the *C. miles* isolated from Australia [15] utilized different sequencing platforms with varying throughputs, making direct comparisons between 454-based and Illumina venom gland transcriptomes challenging. Furthermore, the observed discrepancies may be attributed to intrinsic differences in the reference databases used for transcriptome annotation.

The conopeptides identified in this study comprise a substantial part of the venom library, although they likely represent only a fraction of its full diversity. Fully capturing this variation would require extensive sampling and integration of multi-omics approaches. Nevertheless, analyzing individual specimens also offers significant insights into the venom complexity of *Conus* species.

## 4. Materials and Methods

### 4.1. Sample Collection, Extraction, Sequencing, and Assembly

A specimen of each species was collected in different parts of the Philippines: *C. capitaneus* and *C. mustelinus* in Caw-oy, Cebu, and *C. miles* in Marinduque. The venom ducts of the collected specimens were dissected, stored in RNAlater (Ambion, Austin, TX, USA), and kept at −20 °C until extraction. Venom duct tissue was homogenized using a Precellys 24 tissue homogenizer (Precellys, Bertin Technologies, Montigny-le-Bretonneux, France) in a microcentrifuge tube containing 0.5-mm Zirconia/Silica beads (Biospec Product Inc., Bartlesville, OK, USA). RNA isolation was performed using the Dynabeads mRNA DIRECT Kit (Invitrogen Dynal AS, Oslo, Norway), following the manufacturer’s instructions. The quality of the isolated mRNA was assessed by agarose gel electrophoresis and Qubit assay. Concentrations of mRNA extracted from *C. capitaneus*, *C. miles*, and *C. mustelinus* were determined to be 82.93, 151, and 117.17 ng/µL, respectively, with A260/280 ratios of 1.98, 2.14, and 2.10. Extracted RNA was submitted to the BGI sequencing facility (formerly Beijing Genomics Institute, Shenzhen, China) for cDNA library construction and paired-end sequencing using the Illumina HiSeq 2000 sequencing platform.

Raw sequences were subjected to rcorrector v1.0.4 [20], a k-mer-based corrector using Illumina reads. Adapter trimming, quality filtering, and fine-tuning were conducted using the AfterQC tool v0.9.7 [21], followed by FastQC v0.11.9 [80], to assess the overall quality of reads. Removal of ribosomal RNA (rRNA) sequences was completed by first creating a database of 8S, 18S, and 28S rRNA sequences downloaded from the Silva database (https://www.arb-silva.de/, accessed on 13 January 2023) and then mapped to the reads using Bowtie2 v2.5.1 [81]. Clean reads were assembled using Trinity v2.11.1 [22] with kmer size 25. DETONATE’s RSEM-EVAL v1.8.1 was used to evaluate the quality of the de novo assemblies. This program implements a reference-free evaluation method that relies only on the assembly and the reads used to create it [81].

### 4.2. Putative Conopeptide Prediction

The final transcriptome was subjected to BLAST and profile Hidden Markov Model (pHMM) of the ConoSorter pipeline [9]. ConoSorter predictions were refined based on specific criteria: hydrophobicity of the signal region (>50%), amino acid size of 40, and e-value cut-off of the superfamily (<0.0001) [82]. Additionally, BLASTx similarity searchers of the assembly were run against the reference database conducted with an E-value of 1 × 10^−5^, followed by the translation of selected sequences into amino acids using the universal genetic code [83]. Diamond BLAST v2.1.9 [84], ConoDictor v2.4.1 [85], and multiple sequence alignment using Clustal Omega v1.2.4 (https://www.ebi.ac.uk/Tools/msa/clustalo/, accessed on 18 June 2024) [86] were also used to assign conotoxin candidates to their respective gene superfamilies correctly.

Putative conopeptides were then consolidated into a unified dataset and inspected for duplicates. The conopeptide precursor domains (signal, propeptide, and mature) and cysteine framework were identified using the ConoPrec tool of the ConoServer database [62,87]. Signal sequences were also detected using the signalP 6.0 tool [88]. Conotoxins with complete precursor sequences showing similarity in the pro- or mature region were retained. Highly truncated mature peptide sequences (those that cannot be assigned to the signal and cysteine framework of the mature peptide region) were filtered [89].

To validate gene superfamily assignment, the signal sequences of conopeptides were clustered using a sequence similarity threshold of 75% [19]. Sequences with <75% sequence similarity to any known gene superfamily were tagged as putative novel gene superfamily.

### 4.3. Quantification of Transcript Level Expression

Using RSEM-Bowtie2, transcript expression levels were measured in TPM (transcripts per million) [90]. Only transcripts encoding conotoxins were included in the reference for assessing conotoxin expression levels.

The total expression level was determined by summing the TPM values of all conotoxins and dividing by the total assembly TPM. Conversely, the relative expression level of each gene superfamily was computed by dividing the total superfamily expression by the total conotoxin TPM [25].

Additional conopeptide precursor filtering was applied following the criteria set by Koch [91] and Taguchi [92]. Sequences with TPM values below one were removed to exclude partial or poorly expressed transcripts, potential artifacts, and sequencing errors.

### 4.4. Naming Assignment of Conotoxin Candidates

The predicted conopeptides in this study were referred to as “conotoxin candidates” or “putative conotoxin” until future evidence can verify that a newly identified sequence indeed encodes a biologically active toxin (and is not merely predicted to do so) [93].

Conotoxin precursor sequences from *C. capitaneus* (prefix Cpt), *C. miles* (prefix Mil), and *C. mustelinus* (prefix Mus) were designated following the established conotoxin nomenclature [94]. This nomenclature involves assigning names based on species representation through one to three letters, cysteine framework indicated by an Arabic numeral, and order of discovery denoted by a second numeral following a decimal point. However, to prevent conflicts with previously documented sequences in the ConoServer database for *C. capitaneus* (prefixes Cap, Cp, and Ca), *C. miles* (prefix Mi), and *C. mustelinus* (prefixes Ms and Mt), slight modifications were made, leading to the adoption of the specified prefixes. Superfamilies, whether novel, reclassified, or unclassified, were named based on the initial five amino acids shared by their constituent sequences (e.g., putative MEALT). Finally, as initially reported, the naming conventions used for conopeptides, including those from previously unclassified and potentially new superfamilies, were followed rigorously (e.g., Pmag-02, SF-mi, and Cerm06). These names typically include an abbreviated form of genus or species (e.g., Pmag for *Pionoconus magus*), followed by a numerical identifier that distinguishes individual peptides. The “SF” designation is also used to indicate potentially novel gene superfamilies, as seen in previous reports [15,19].

### 4.5. Assignment of Cysteine Framework Patterns

The assignment of cysteine frameworks is guided by criteria outlined in a study on *C. litteratus* [95], establishing a systematic approach to categorizing these structural motifs based on their composition and cysteine residue distribution. In their scheme, the sequences are classified as cysteine-free (sequences that do not contain any cysteine residues, hence lack of disulfide bridges), No S-S (sequences with a single cysteine residue, denoted as C1, and the presence of cysteine without disulfide bonds), 1 S-S (sequences that have either two cysteines that are either adjacent (-C-C-) or separated by one or more amino acids (-C-X-C-) and connected by a single disulfide bond), canonical cysteine types (sequences with established patterns of cysteine distribution that have been recognized and documented), and Unknown (sequences with an even number of cysteine residues that do not fit into the established canonical types, indicating the potential for novel cysteine frameworks).

### 4.6. Shannon’s Diversity Index of Venom Conopeptides

The diversity within the *C. capitaneus*, *C. miles*, and *C. mustelinus* conopeptide datasets were measured using Shannon’s diversity index, denoted as *H*’,

$$H^{\prime }=\sum_{i=1}^{S}pi(lnpi){\;\;\;\;\;\;E}_{H^{\prime }}=H/lnS$$

where *S* represents the count of conopeptide gene superfamilies, and pi reflects the fraction of conopeptides associated with the ith superfamily within the dataset [44,63,96]. Additionally, the evenness of the conopeptide datasets was assessed through Shannon’s equitability, expressed as $E_{H^{\prime }}$, with *S* indicating the richness of the dataset, determined by the total number of conopeptide gene superfamilies. Computation was performed using the Vegan R Bioconductor package [97].

## 5. Conclusions

This study explored the complexity of the venom repertoire in three closely related *Rhizoconus* species, presenting the first venom gland transcriptome assemblies of *C. capitaneus* and *C. mustelinus*. Additionally, the venom gland transcriptome of *C. miles* revealed new gene superfamilies, adding to the gene superfamily diversity of the previously studied *C. miles* transcriptome from Australia. A total of 696 putative conopeptides were identified: 225 in *C. capitaneus*, 121 in *C. miles*, and 168 in *C. mustelinus*. These conopeptides span 29 canonical gene superfamilies, several minor and divergent gene superfamilies, conopeptide classes including hormone-like conopeptides, and 12 potentially novel gene superfamilies. The transcriptomes of *Rhizoconus* species demonstrate high diversity in gene superfamilies, with D, B2, G2, and I2 showing high expression levels. The analysis identified putative novel conopeptides with sequence similarity to the previously characterized αD-conotoxins from the *Rhizoconus* species, a somatostatin analog consomatin Ro1, and granulin-like conopeptides identified in the *C. miles* venom gland transcriptome. These findings offer insights into the conotoxin cocktails employed by *Rhizoconus* species for prey capture and defense. Further studies, including additional sample sequencing and an integrated multi-omics approach, are essential to fully capture the genetic diversity and variation in the venom peptides within the species and to further validate the study’s findings. Nonetheless, the study provides a foundation for future research focusing on *Rhizoconus* species to study their biology, evolution, and potential as sources of drug leads.

## Figures and Tables

**Table 1:** Summary of statistics of the transcriptome assembly.

	*C. capitaneus*	*C. miles*	*C. mustelinus*
Number of transcripts	133,698	56,751	77,764
Mean length (bp)	599.38	480.46	538.21
Number of contigs >1 kb	18,029	4514	8333
N50	801	540	664
RSEM% alignment rate	81.48	88.84	87.57
DETONATE score (×10^9^)	−2.269	−1.171	−2.168

**Table 2:** Diversity of conopeptides in the studied *Conus* species.

Species	Number of Superfamilies	Unique Conopeptide Sequences	Shannon Diversity Index	Evenness
*C. capitaneus*	53	225	3.30	0.73
*C. miles*	48	121	3.43	0.76
*C. mustelinus*	45	168	3.04	0.78

**Table 3:** Summary of conopeptide abundance and relative gene expression from different *Conus* species.

Species	Diet	No. of Unique Conopeptide Precursors	% Total TPM Attributed to Conopeptides	Sequencing Platform	Reference
*C. capitaneus*	V	225	19.0%	Illumina	This study
*C. miles*	V	121	56.1%		
*C. mustelinus*	V	168	51.5%		
*C. rattus*	V	102	35.5%	Illumina	[25]
*C. quercinus*	V	97	49.5%		
*C. californicus*	G	185	26.0%		
*C. geographus*	P	136	9.33%	Illumina	[26]
*C. rolani*	P	110	13.96%		
*C. striatus*	P	212	43.71%		

V—vermivorous; P—piscivorous; G—generalist.

**Table 4:** Transcripts per million (TPM) values for hormone-like conopeptides identified in this study.

Hormone-like Conopeptides	TPM Values		
	*C. capitaneus*	*C. miles*	*C. mustelinus*
C/Consomatin	4.96	8.94	24.15
Insulin	20.76	9.08	599.34
Conopressin/conophysin	5.41	33.5	1.24
G2/Granulin-like	-	32,050.46	-
Thyrostimulin	1.46	-	-
Prohormone-4	-	-	142.69

- Not identified.

**Table 5:** Conopeptides (mature region) predicted in this study, identical in sequence to previously reported peptides in the *C. miles* proteome confirmed by MS/MS [19].

Protein Sequence (MS/MS >99% Confidence)	*C. miles* ID [19]	Superfamily	ID of Identical Peptides in *C. miles* Transcriptome in this Study	TPM
ECREKGQGCTNTALCCPGLECEGQSQGGLCVDN	Mi001	O1	Mil078.O1; Mil082.O1	424,403.49; 1070.07
GGGCSQHPHCCGGTCNK	Mi023	O1	Mil084.O1	198
CTDDSQFCNPSNHDCCSGKCIDEGDNGICAIVPENS	Mi027	O1	Mil081.O1	1091.49
CPNLTCKCSGSPLCTRYRCKT	Mi035	O2	Mil1096.O2	11,896.05
CKCTSAPDCNFYKCRT	Mi036	O2	Mil097.O2; Mil099.O2	5218.27; 1969.75
DCCSLSACVPPPACECCK	Mi040	M	Mil061.M	6763.05
SSCPPACCPTC	Mi041	L	Mil059.L	355.09
CCPKKPYCCPG	Mi042	T	Mil118.T	40.53
VPCQQGGGK	Mi043	I2	Mil052.I2	99.93
CMPCGGECCCEPNSCIDGTCHHE	Mi045	G2 (Granulin-like)	Mil046.G2	32,050.46
MLKVGVVFLVFLVLLSLADSWNGDNPGRQRGEKQSPQRNVFRSNLRKYNSYQKRRCANSTPCGECTDEGKICQVQPGGKGTCGECVPNTR	Mi040	M	Mil062.M	258.31
MSKTGLVLVVLYLLSSPVNLQQNEDDQAFSKIETRDRPECYNCFPNDDGHCVGTCCGEDSCKGGIRGCGCL	Mi044	SF-mi1	Mil113.SF-mi1	20.35

## Data Availability

All the original Illumina raw read files were submitted to the NCBI archive under Bioproject PRJNA1211556.

## Supplementary Materials

The following supporting information can be downloaded at https://www.mdpi.com/article/10.3390/md23070266/s1. Supplementary File S1: Metadata; Supplementary File S2: Multiple sequence alignment of identified conopeptides; Supplementary File S3: *C. miles* comparison; Supplementary File S4: Relative expression; Supplementary File S5: Abundance and expression levels.

## References

[1] Olivera B.M. (2006). *Conus* Peptides: Biodiversity-based Discovery and Exogenomics. J. Biol. Chem..

[2] Zhao Y., Antunes A. (2022). Biomedical Potential of the Neglected Molluscivorous and Vermivorous *Conus* Species. Mar. Drugs.

[3] Aman J.W., Imperial J.S., Ueberheide B., Zhang M.M., Aguilar M., Taylor D., Watkins M., Yoshikami D., Showers-Corneli P., Safavi-Hemami H. (2015). Insights into the Origins Of Fish Hunting in Venomous Cone Snails from Studies of *Conus Tessulatus*. Proc. Natl. Acad. Sci. USA.

[4] Duda T.F., Kohn A.J. (2005). Species-Level Phylogeography and Evolutionary History of the Hyperdiverse Marine Gastropod Genus *Conus*. Mol. Phylogenet. Evol..

[5] Imperial J., Silverton N., Olivera B.M., Bandyopadhay P., Sporning A., Ferber M., Terlau H. (2017). Using Chemistry to Reconstruct Evolution: On the Origins of Fish-Hunting in Venomous Cone Snails. Proc. Am. Philos. Soc..

[6] Duda T.F., Kohn A.J., Palumbi S.R. (2001). Origins of Diverse Feeding Ecologies within *Conus*, a Genus of Venomous Marine Gastropods. Biol. J. Linn. Soc..

[7] Olivera B.M., Showers-Corneli P., Watkins M., Fedosov A. (2014). Biodiversity of Cone Snails and Other Venomous Marine Gastropods: Evolutionary Success through Neuropharmacology. Annu. Rev. Anim. Biosci..

[8] Akondi K.B., Muttenthaler M., Dutertre S., Kaas Q., Craik D.J., Lewis R.J., Alewood P.F. (2014). Discovery, Synthesis, and Structure-activity Relationships of Conotoxins. Chem. Rev..

[9] Lavergne V., Dutertre S., Jin A.H., Lewis R.J., Taft R.J., Alewood P.F. (2013). Systematic Interrogation of the *Conus marmoreus* Venom Duct transcriptome with ConoSorter reveals 158 Novel Conotoxins and 13 New Gene Superfamilies. BMC Genom..

[10] Olivera B.M., Watkins M., Bandyopadhyay P., Imperial J.S., de la Cotera E.P., Aguilar M.B., Vera E.L., Concepcion G.P., Lluisma A. (2012). Adaptive Radiation of Venomous Marine Snail Lineages and the Accelerated Evolution of Venom Peptide Genes. Ann. N. Y. Acad. Sci..

[11] Terlau H., Olivera B.M. (2004). *Conus* venoms: A Rich Source of Novel Ion Channel-Targeted Peptides. Physiol. Rev..

[12] Fu Y., Li C., Dong S., Wu Y., Zhangsun D., Luo S. (2018). Discovery Methodology of Novel Conotoxins from *Conus* species. Mar. Drugs.

[13] Robinson S.D., Li Q., Lu A., Bandyopadhyay P.K., Yandell M., Olivera B.M., Safavi-Hemami H. (2017). The Venom Repertoire of *Conus gloriamaris* (Chemnitz, 1777), the Glory of the Sea. Mar. Drugs.

[14] Nachtigall P.G., Rautsaw R.M., Ellsworth S.A., Mason A.J., Rokyta D.R., Parkinson C.L., Junqueira-De-Azevedo I.L.M. (2021). ToxCodAn: A New Toxin Annotator and Guide to Venom Gland Transcriptomics. Brief. Bioinform..

[15] Jin A.H., Dutertre S., Kaas Q., Lavergne V., Kubala P., Lewis R.J., Alewood P.F. (2013). Transcriptomic Messiness in the Venom Duct of *Conus miles* Contributes to Conotoxin Diversity. Mol. Cell. Proteom..

[16] Zhang H., Fu Y., Wang L., Liang A., Chen S., Xu A. (2019). Identifying Novel Conopepetides from the Venom Ducts of *Conus litteratus* through Integrating Transcriptomics and Proteomics. J. Proteom..

[17] Abalde S., Tenorio M.J., Afonso C.M.L., Zardoya R. (2020). Comparative Transcriptomics of the Venoms of Continental and Insular Radiations of West African Cones. Proc. R. Soc. B.

[18] Dutt M., Dutertre S., Jin A.H., Lavergne V., Alewood P.F., Lewis R.J. (2019). Venomics Reveals Venom Complexity of the Piscivorous Cone Snail, *Conus tulipa*. Mar. Drugs.

[19] Jin A.H., Dutertre S., Dutt M., Lavergne V., Jones A., Lewis R.J., Alewood P.F. (2019). Transcriptomic-Proteomic Correlation in the Predation-Evoked Venom of the Cone Snail, *Conus imperialis*. Mar. Drugs.

[20] Song L., Florea L. (2015). Rcorrector: Efficient and Accurate Error Correction for Illumina RNA-seq reads. GigaScience.

[21] Chen S., Huang T., Zhou Y., Han Y., Xu M., Gu J. (2017). AfterQC: Automatic Filtering, Trimming, Error Removing and Quality Control for Fastq Data. BMC Bioinform..

[22] Grabherr M.G., Haas B.J., Yassour M., Levin J.Z., Thompson D.A., Amit I., Adiconis X., Fan L., Raychowdhury R., Zeng Q. (2011). Full-Length Transcriptome Assembly from RNA-Seq Data Without a Reference Genome. Nat. Biotechnol..

[23] Jiang H., Wang C.Z., Xu C.Q., Fan C.X., Dai X.D., Chen J.S., Chi C.W. (2006). A Novel M-superfamily Conotoxin with a Unique Motif from *Conus vexillum*. Peptides.

[24] Robinson S.D., Safavi-Hemami H., McIntosh L.D., Purcell A.W., Norton R.S., Papenfuss A.T. (2014). Diversity of Conotoxin Gene Superfamilies in the Venomous Snail, *Conus victoriae*. PLoS ONE.

[25] Phuong M.A., Mahardika G.N., Alfaro M.E. (2016). Dietary Breadth is Positively Correlated with Venom Complexity in Cone Snails. BMC Genom..

[26] Fedosov A., Tucci C.F., Kantor Y., Farhat S., Puillandre N. (2023). Collaborative Expression: Transcriptomics of *Conus virgo* Suggests Contribution of Multiple Secretory Glands to Venom Production. J. Mol. Evol..

[27] Dutertre S., Jin A.H., Kaas Q., Jones A., Alewood P.F., Lewis R.J. (2013). Deep Venomics Reveals the Mechanism for Expanded Peptide Diversity in Cone Snail Venom. Mol. Cell. Proteom..

[28] Kauferstein S., Kendel Y., Nicke A., Coronas F.I.V., Possani L.D., Favreau P., Križaj I., Wunder C., Kauert G., Mebs D. (2009). New Conopeptides of the D-Superfamily Selectively Inhibiting Neuronal Nicotinic Acetylcholine Receptors. Toxicon.

[29] Loughnan M.L., Nicke A., Lawrence N., Lewis R.J. (2009). Novel Alpha D-Conopeptides and their Precursors Identified By cDNA Cloning Define the D-Conotoxin Superfamily. Biochemistry.

[30] Prashanth J.R., Dutertre S., Jin A.H., Lavergne V., Hamilton B., Cardoso F.C., Griffin J., Venter D.J., Alewood P.F., Lewis R.J. (2016). The Role of Defensive Ecological Interactions in the Evolution of Conotoxins. Mol. Ecol..

[31] Luo S., Zhangsun D., Feng J., Wu Y., Zhu X., Hu Y. (2007). Diversity of the O-superfamily conotoxins from *Conus miles*. J. Pept. Sci..

[32] Ramiro I.B.L., Bjørn-Yoshimoto W.E., Imperial J.S., Gajewiak J., Florez Salcedo P., Watkins M., Taylor D., Resager W., Ueberheide B., Brauner-Osborne H. (2022). Somatostatin Venom Analogs Evolved by Fish-Hunting Cone Snails: From Prey Capture Behavior to Identifying Drug Leads. Sci. Adv..

[33] Jin A.H., Dekan Z., Smout M.J., Wilson D., Dutertre S., Vetter I., Lewis R.J., Loukas A., Daly N.L., Alewood P.F. (2017). Conotoxin Φ-MiXXVIIA from the Superfamily G2 Employs a Novel Cysteine Framework that Mimics Granulin and Displays Anti-Apoptotic Activity. Angew. Chem. Int. Ed..

[34] Olivera B.M. (2002). *Conus* Venom Peptides: Reflections from the Biology of Clades and Species. Annu. Rev. Ecol. Syst..

[35] Kohn A.J. (2001). Maximal Species Richness in *Conus*: Diversity, Diet and Habitat on Reefs of Northeast Papua New Guinea. Coral Reefs.

[36] Elliger C.A., Richmond T.A., Lebaric Z.N., Pierce N.T., Sweedler J.V., Gilly W.F. (2011). Diversity of Conotoxin Types from *Conus californicus* Reflects a Diversity of Prey Types and a Novel Evolutionary History. Toxicon.

[37] Remigio E.A., Duda T.F. (2008). Evolution of Ecological Specialization and Venom of a Predatory Marine Gastropod. Mol. Ecol..

[38] Himaya S.W.A., Arkhipov A., Yum W.Y., Lewis R.J. (2022). Comparative Venomics of *C. flavidus* and *C. frigidus* and Closely Related Vermivorous Cone Snails. Mar. Drugs.

[39] Lewis R.J., Dutertre S., Vetter I., Christie M.J. (2012). *Conus* Venom Peptide Pharmacology. Pharmacol. Rev..

[40] Turner A., Kaas Q., Craik D.J. (2020). Hormone-Like Conopeptides—New Tools for Pharmaceutical Design. RSC Med. Chem..

[41] Olivera B.M., Seger J., Horvath M.P., Fedosov A.E. (2015). Prey-capture Strategies of Fish-hunting Cone Snails: Behavior, neurobiology and evolution. Brain Behav. Evol..

[42] Safavi-Hemami H., Hu H., Gorasia D.G., Bandyopadhyay P.K., Veith P.D., Young N.D., Reynolds E.C., Yandell M., Olivera B.M., Purcell A.W. (2014). Combined Proteomic and Transcriptomic Interrogation of the Venom Gland of *Conus geographus* Uncovers Novel Components and Functional Compartmentalization. Mol. Cell. Proteom..

[43] Robinson S., Norton R. (2014). Conotoxin gene superfamilies. Mar. Drugs.

[44] Barghi N., Concepcion G.P., Olivera B.M., Lluisma A.O. (2015). Comparison of the Venom Peptides and Their Expression in Closely Related *Conus* Species: Insights into Adaptive Post-Speciation Evolution of *Conus* Exogenomes. Genome Biol. Evol..

[45] Yao G., Peng C., Zhu Y., Fan C., Jiang H., Chen J., Cao Y., Shi Q. (2019). High-Throughput Identification and Analysis of Novel Conotoxins from Three Vermivorous Cone Snails by Transcriptome Sequencing. Mar. Drugs.

[46] Loughnan M., Nicke A., Jones A., Schroeder C.I., Nevin S.T., Adams D.J., Alewood P.F., Lewis R.J. (2006). Identification of a Novel Class of Nicotinic Receptor Antagonists: Dimeric Conotoxins VxXIIA, VxXIIB, and VxXIIC from *Conus vexillum*. J. Biol. Chem..

[47] Xu S., Zhang T., Kompella S.N., Yan M., Lu A., Wang Y., Shao X., Chi C., Adams D.J., Ding J. (2015). Conotoxin αD-GeXXA Utilizes a Novel Strategy to Antagonize Nicotinic Acetylcholine Receptors. Sci. Rep..

[48] Hernández-Sámano A.C., Falcón A., Zamudio F., Batista C.V., Michel-Morfín J.E., Landa-Jaime V., López-Vera E., Jeziorski M.C., Aguilar M.B. (2019). αD-Conotoxins in Species of the Eastern Pacific: The Case of *Conus princeps* from Mexico. Toxins.

[49] Rodriguez-Ruiz X.C., Aguilar M.B., Ortíz-Arellano M.A., Safavi-Hemami H., López-Vera E. (2022). A Novel Dimeric Conotoxin, FrXXA, from the Vermivorous Cone Snail *Conus fergusoni*, of the Eastern Pacific, Inhibits Nicotinic Acetylcholine Receptors. Toxins.

[50] Decker M.W., Rueter L.E., Bitner R.S. (2004). Nicotinic Acetylcholine Receptor Agonists: A Potential New Class of Analgesics. Curr. Top. Med. Chem..

[51] Giribaldi J., Dutertre S. (2018). α-Conotoxins to Explore the Molecular, Physiological and Pathophysiological Functions of Neuronal Nicotinic Acetylcholine Receptors. Neurosci. Lett..

[52] Craig A.G. (2000). The Characterization of Conotoxins. J. Toxicol. Toxin Rev..

[53] Mebs D., Kordiš D., Kendel Y., Kauferstein S. (2011). The Evolution of αD-Conopeptides Targeting Neuronal Nicotinic Acetylcholine Receptors. Acta Chim. Slov..

[54] Ratibou Z., Inguimbert N., Dutertre S. (2024). Predatory and Defensive Strategies in Cone Snails. Toxins.

[55] Lluisma A.O., Milash B.A., Moore B., Olivera B.M., Bandyopadhyay P.K. (2012). Novel Venom Peptides from the Cone Snail *Conus Pulicarius* Discovered Through Next-Generation Sequencing of its Venom Duct Transcriptome. Mar. Genom..

[56] Li Q., Barghi N., Lu A., Fedosov A.E., Bandyopadhyay P.K., Lluisma A.O., Concepcion G.P., Yandell M., Olivera B.M., Safavi-Hemami H. (2017). Divergence of the Venom Exogene Repertoire in Two Sister Species of *Turriconus*. Genome Biol. Evol..

[57] Yuan D.D., Han Y.H., Wang C.G., Chi C.W. (2007). From the Identification of Gene Organization of Alpha Conotoxins to the Cloning of Novel Toxins. Toxicon.

[58] Puillandre N., Bouchet P., Duda T., Kauferstein S., Kohn A., Olivera B., Watkins M., Meyer C. (2014). Molecular phylogeny and evolution of the cone snails (Gastropoda, Conoidea). Mol. Phylogenetics Evol..

[59] Whiteaker P., Christensen S., Yoshikami D., Dowell C., Watkins M., Gulyas J., Rivier J., Olivera B.M., McIntosh J.M. (2007). Discovery, Synthesis, and Structure Activity of a Highly Selective α7 Nicotinic Acetylcholine Receptor Antagonist. Biochemistry.

[60] Wu Y., Wang L., Zhou M., You Y., Zhu X., Qiang Y., Qin M., Luo S., Ren Z., Xu A. (2013). Molecular Evolution and Diversity of *Conus* Peptide Toxins, as Revealed by Gene Structure and Intron Sequence Analyses. PLoS ONE.

[61] Franco A., Kompella S.N., Akondi K.B., Melaun C., Daly N.L., Luetje C.W., Alewood P.F., Craik D.J., Adams D.J., Marí F. (2012). RegIIA: An α4/7-Conotoxin from the Venom of *Conus regius* that Potently Blocks α3β4 nAChRs. Biochem. Pharmacol..

[62] Kaas Q., Westermann J., Craik D.J. (2010). Conopeptide characterization and classifications: An analysis using ConoServer. Toxicon.

[63] Lu A., Watkins M., Li Q., Robinson S.D., Concepcion G.P., Yandell M., Weng Z., Olivera B.M., Safavi-Hemami H., Fedosov A.E. (2020). Transcriptomic Profiling Reveals Extraordinary Diversity of Venom Peptides in Unexplored Predatory Gastropods of the Genus *Clavus*. Genome Biol. Evol..

[64] Koch T.L., Ramiro I.B.L., Salcedo P.F., Engholm E., Jensen K.J., Chase K., Olivera B.M., Bjørn-Yoshimoto W.E., Safavi-Hemami H. (2022). Reconstructing the Origins of the Somatostatin and Allatostatin-C signaling Systems Using the Accelerated Evolution of Biodiverse Cone Snail Toxins. Mol. Biol. Evol..

[65] Bateman A., Bennett H.P. (2009). The Granulin Gene Family: From Cancer to Dementia. BioEssays News Rev. Mol. Cell. Dev. Biol..

[66] Jacobsen R.B., Jimenez E.C., De la Cruz R.G.C., Gray W.R., Cruz L.J., Olivera B.M. (1999). A Novel D-Leucine-Containing *Conus* Peptide: Diverse Conformational Dynamics in the Contryphan Family. J. Pept. Res..

[67] Rodriguez A.M., Dutertre S., Lewis R.J., Marí F. (2015). Intraspecific Variations in *Conus purpurascens* Injected Venom Using LC/MALDI-TOF-MS and LC-ESI-TripleTOF-MS. Anal. Bioanal. Chem..

[68] Chun J.B.S., Baker M.R., Kim D.H., LeRoy M., Toribo P., Bingham J.P. (2012). Cone Snail Milked Venom Dynamics—A Quantitative Study of *Conus purpurascens*. Toxicon Off. J. Int. Soc. Toxinology.

[69] Möller C., Marí F. (2011). 9.3 kDa Components of the Injected Venom of *Conus purpurascens* Define a New 5-disulfide Conotoxin Framework. Biopolymers.

[70] Grandal M., Hoggard M., Neely B., Davis W.C., Marí F. (2021). Proteogenomic Assessment of Intraspecific Venom Variability: Molecular Adaptations in the Venom Arsenal of *Conus purpurascens*. Mol. Cell. Proteom..

[71] Safavi-Hemami H., Siero W.A., Gorasia D.G., Young N.D., MacMillan D., Williamson N.A., Purcell A.W. (2011). Specialisation of the Venom Gland Proteome in Predatory Cone Snails Reveals Functional Diversification of the Conotoxin Biosynthetic Pathway. J. Proteome Res..

[72] Chang D., Olenzek A.M., Duda T.F. (2015). Effects of Geographical Heterogeneity in Species Interactions on the Evolution of Venom Genes. Proc. Biol. Sci..

[73] Gao B., Peng C., Zhu Y., Sun Y., Zhao T., Huang Y., Shi Q. (2018). High Throughput Identification of Novel Conotoxins from the Vermivorous Oak Cone Snail (*Conus quercinus*) by Transcriptome Sequencing. Int. J. Mol. Sci..

[74] Dutertre S., Jin A., Vetter I., Hamilton B., Sunagar K., Lavergne V., Dutertre V., Fry B.G., Antunes A., Venter D.J. (2014). Evolution of Separate Predation- and Defence-Evoked Venoms in Carnivorous Cone Snails. Nat. Commun..

[75] Abdel-Rahman M.A., Abdel-Nabi I.M., El-Naggar M.S., Abbas O.A., Strong P.N. (2011). Intraspecific Variation in the Venom of the Vermivorous Cone Snail *Conus vexillum*. Comp. Biochem. Physiol. Part C Toxicol. Pharmacol..

[76] Prator C.A., Murayama K.M., Schulz J.R. (2014). Venom Variation during Prey Capture by the Cone Snail, *Conus textile*. PLoS ONE.

[77] Davis J., Jones A., Lewis R.J. (2009). Remarkable Inter- and Intraspecies Complexity of Conotoxins Revealed by LC/MS. Peptides.

[78] Dutertre S., Biass D., Stocklin R., Favreau P. (2010). Dramatic Intraspecimen Variations Within the Injected Venom of Conus consors: An Unsuspected Contribution to Venom Diversity. Toxicon.

[79] Jakubowski J.A., Kelley W.P., Sweedler J.V., Gilly W.F., Schulz J.R. (2005). Intraspecific Variation of Venom Injected by Fish-hunting *Conus* snails. J. Exp. Biol..

[80] Andrews S. (2010). FastQC: A Quality Control Tool for High Throughput Sequence Data. http://www.bioinformatics.babraham.ac.uk/projects/fastqc.

[81] Langmead B., Salzberg S. (2012). Fast gapped-read alignment with Bowtie 2. Nat. Methods.

[82] Pardos-Blas J.R., Irisarri I., Abalde S., Tenorio M.J., Zardoya R. (2019). Conotoxin Diversity in the Venom Gland Transcriptome of the Magician’s Cone, *Pionoconus magus*. Mar. Drugs.

[83] Prashanth J.R., Lewis R.J. (2015). An Efficient Transcriptome Analysis Pipeline to Accelerate Venom Peptide Discovery and Characterisation. Toxicon.

[84] Buchfink B., Xie C., Huson D.H. (2014). Fast and Sensitive Protein Alignment using DIAMOND. Nat. Methods.

[85] Koua D., Ebou A., Dutertre S. (2021). Improved Prediction of Conopeptide Superfamilies with ConoDictor 2.0. Bioinform. Adv..

[86] Madeira F., Pearce M., Tivey A.R.N., Basutkar P., Lee J., Edbali O., Madhusoodanan N., Kolesnikov A., Lopez R. (2022). Search and Sequence Analysis Tools Services from EMBL-EBI in 2022. Nucleic Acids Res..

[87] Kaas Q., Yu R., Jin A.H., Dutertre S., Craik D.J. (2012). ConoServer: Updated Content, Knowledge, and Discovery Tools in the Conopeptide Database. Nucleic Acids Res..

[88] Teufel F., Almagro Armenteros J.J., Johansen A.R., Gíslason M.H., Pihl S.I., Tsirigos K.D., Winther O., Brunak S., von Heijne G., Nielsen H. (2022). SignalP 6.0 Predicts All Five Types of Signal Peptides using Protein Language Models. Nat. Biotechnol..

[89] Abalde S., Tenorio M.J., Afonso C.M.L., Zardoya R. (2018). Conotoxin Diversity in *Chelyconus ermineus* (Born, 1778) and the Convergent Origin of Piscivory in the Atlantic and Indo-Pacific Cones. Genome Biol. Evol..

[90] Li B., Dewey C.N. (2011). RSEM: Accurate Transcript Quantification from RNA-Seq data With or Without A Reference Genome. BMC Bioinform..

[91] Koch T.L., Robinson S.D., Salcedo P.F., Chase K., Biggs J., Fedosov A.E., Yandell M., Olivera B.M., Safavi-Hemami H. (2024). Prey Shifts Drive Venom Evolution in Cone Snails. Mol. Biol. Evol..

[92] Taguchi R., Masacupan D.J., Lluisma A. (2024). Diversity and Novelty of Venom Peptides from *Conus (Asprella) rolani* Revealed by Analysis of its Venom Duct Transcriptome. SciEnggJ.

[93] Bjørn-Yoshimoto W.E., Ramiro I.B.L., Yandell M., McIntosh J.M., Olivera B.M., Ellgaard L., Safavi-Hemami H. (2020). Curses or Cures: A Review of the Numerous Benefits Versus the Biosecurity Concerns of Conotoxin Research. Biomedicines.

[94] Walker C.S., Steel D., Jacobsen R.B., Lirazan M.B., Cruz L.J., Hooper D., Shetty R., DelaCruz R.C., Nielsen J.S., Zhou L.M. (1999). The T-superfamily of Conotoxins. J. Biol. Chem..

[95] Li X., Chen W., Zhangsun D., Luo S. (2020). Diversity of Conopeptides and Their Precursor Genes of *Conus litteratus*. Mar. Drugs.

[96] Fassio G., Modica M.V., Mary L., Zaharias P., Fedosov A.E., Gorson J., Kantor Y.I., Holford M., Puillandre N. (2019). Venom Diversity and Evolution in the Most Divergent Cone Snail Genus *Profundiconus*. Toxins.

[97] Oksanen J., Simpson G., Blanchet F., Kindt R., Legendre P., Minchin P., O’Hara R., Solymos P., Stevens M., Szoecs E. Vegan: Community Ecology Package. https://CRAN.R-project.org/package=vegan.

