# Supplementary material for: Diversity and Novelty of Venom Peptides in Vermivorous Cone Snails, Subgenus Rhizoconus (Gastropoda: Mollusca)
Source: Mar Drugs. 2025 Jun 26;23(7):266. doi: 10.3390/md23070266 (PMC12300008; doi:10.3390/md23070266)
Supplement: Supplementary file 1 [file marinedrugs-23-00266-s001.zip › Supplementary Files/Supplementary File S2. Multiple sequence alignment of identified conopeptides.pdf]

Multiple Sequence Alignment

```
A-Superfamily
Cpt003.A      MGMRTMFTVFLLVVLATTVVS---DRASNRENRRASN-----WNTRIAYIGCCDIPDCYNKNREQCLDESSG--------------        64
Mil003.A      MGMRMMFTVFLLVVLATTVVSFIFDRTYEDVGGPPKLY----TDEGLTEDQCCQYAACWRVESVIC--------------------        62
Mil004.A      -------TVFLLVVLATTVVSSNSDRDPALGGRNAAAIASDKIASTLRRRGCCSHPACSVNHPEMCGRR-----------------        62
Cpt001.A      MGMQMMFTVFLLVVLATTVVSIPSDRASDGRNAVVHERAPELVVTAT--TNC-----CGYNPMTICPPCMCTYSCPPKRKPGRRND        79
Mil005.A      MGMRMMFTVFLLVVLATTVVSIPSDRASDGRNAAVNERQTWLVPSTI--TTC-----CGYSPGTMCPPCMCTNTC-----------        68
Mil001.A      MGMRMMFTVFLLVVLATTVVSIPSDRASDGRNAAANEKASDVIALAL--KGCCSNPVCHLEHSNMCGRRR----------------        68
Mil002.A      MGMRMMFTVFLLVVLATTVVSIPSDRASDGRDDEAKDERSDMHESGRKGRGRCCHPACGPNY--SCGRCSRTL-------------        71
Cpt002.A      MGMQMMFTVFLLVVLTTTVVSFPSDRASDGRDDEAKDERSDMYESKR--NGRCCHPACGKNY--SCGR------------------        64
Mus001.A      MGMQMMFTVFLLVVLATTVVSIPSDRASDGRDDEAKDERSDMYESKR--NGRCCHPACGKNY--SCGR------------------        64
B1-Superfamily
Cpt005.B1     MQLYTYLYLLVPLVTFHLILGTGTPAHGGALTERRSADATALKPEPVLLQKSAARSTDGNGKDRLTQRKRILKKRGNKARGWEEDREIAETVRELEEIGRR------------------------------  101
Cpt004.B1     MQLYTYLYLLVPLVTFHLILGTGTPAHGGALTERRSADATALKPEPVLLQKSAARSTDGNGKDRLTQRKRILKKRGNAARDAEEVRESAETLHEISVYKKRINQAGCSTHSSVLKSPDRSYPVFATFFLSL  131
Mus002.B1     ------LYLLVPLVTFHLILGTGTLAHGGALTERRSADATALKPEPVLLQKSAARSTDGNGKDRLTQRKRILKKRGNAARDAEEVRESAETLHEISVYKKRINQAGCSTHSSVLKSPDRSYPVFATFFLSL  125
Mil006.B1     MQLYTYLYLLVPLVTFHLILGTGTLDHGGALTERRSTDATALKPEPVLLQKSSARSTDDNGKD--TQMKRILKKRGNKARGEEEVSENAVEFARELARKR-------------------------------  98
Mil007.B1     MQLYTYLYLLVPLVTFHLILGTGTLDHGGALTERRSTDATALKPEPVLLQKSSARSTDDNGKD--TQMKRILKKRGNKARDDEEYAEFIE-GEREAGRTS-----SWVFHVTLVSSK---VPR--------  112
B2-Superfamily
Mil010.B2      ------------------------------------------------------------         0
Mil008.B2      MLRLVIAAVLVCACLAFPQRREGAPAD--NLKSFDPGM------MQGMGGAMPNMQPMQP         52
Mil009.B2      MLRLVIAAVLVCACLAFPQRREGAPAD--NLKSFDPGM------MQGMGGAMPNMQPMQP         52
Mus004.B2      MLRLVIAAVLVCACLAFPQRREGAQAD--NLKPFDP--------MQGMSGGMPNMQGMQP         50
Cpt006.B2      MLRLVIAAVLVCACLAFPQRREGAQAD--NLKSFDPAM------MQGMTGGMPNMQAMQP         52
Cpt008.B2      MLRLVIAAVLVCACLAFPQRREGAQAD--NLKSFDPAM------MQGMTGGMPNMQAMQP         52
Mil011.B2      MLRLIIAAVLATACLAFPQRRDGLPGEAANLKPFGPDMQGMQAMPGAMPGPMPNMQPMQA         60
Cpt007.B2      MLRLIIAAVLASACLAYPQKRDGAPADSANLQPFDAGMQAMPAMP--------NMQGMQG         52
Mus003.B2      MLRLIIAAVLASACLAYPQKRDGAPADSANLQPFDAGMQAMPAMP--------NMQGMQG         52
Mil012.B2      MLRLIIAAVLATACLAYPQRRDGAPADAANLQSFDPGMQAMPGMP--------NMQAMQG         52

Mil010.B2      ----------------NLGMGFKRAADENLEKRKHHSKFQDENKSPFDSPADSLLGNFNFGKFLQENPDNIPFANMENANPGNLGNFEPNAEDSKEGHFRFFDQQQ   90
Mil008.B2      MS-PGMAAGQMLPFNPNMALGFR-SL-ENLENRKHHSKFNEDNKSPFDAP-DADLEKFNFANFLKE----------------------------------------   114
Mil009.B2      MS-PGMAAGQMLPFNPNMALGFR-----------------------------------------------------------------------------------   74
Mus004.B2      MA-GNMAPGQMLPFNPNMALGFKRSL-ENLEKRRHHVNFNEDNKIPFEAP-DADLEKFNFANFLKD----------------------------------------   113
Cpt006.B2      MA-GNMAPGQMLPFNPNMALGFKRSL-ENLEKRRHHSKFNEDNKIPFEAP-DADLEKFNFANFLKD----------------------------------------   115
Cpt008.B2      MA-GNMAPGQMLPFNPNMALGFKR----------------------------------------------------------------------------------   75
Mil011.B2      MP------GQFLPFN-------------------------------------------------------------------------------------------   69
Cpt007.B2      MPMPGIA-SQFLPFNPNLGLGYRRDVDENLEKRKHHSKFNEDNKSPFSAE-DGLG---NFMNFMKENGNNLPFAQMDNGAP-DLGNFEPSAEK-EDGKFRFFDEQQ   151
Mus003.B2      MPMPGIA-SQFLPFNPNLGLGYRRDVDENLEKRKHHSKFNEDNKSPFSAE-DGLG---NFMNFMKENGNNLPFAQMDNGAP-DLGNFEPSAEK-EDGKFRFFDEQQ   151
Mil012.B2      MPMQAMAGGQFLPFNPNFGMGYRRDVDENLEKRRQHSQFNADKSSF--DS-DSLG---NIMDFMNENGNSLPFANMDSAAT-DLGNFEPSAEN-EDGKFRFFDKQQ   150
C/Consomatin-Superfamily
Mus006.C      MKTAYWMMVMMMVVGFTVGGHVHQSHSPTSRSHGDDSIHDKTIHQHLFARLPLENNDDHRSVDLPAGVGADGMKLQRQRRQDFCC-------------------- 85
Cpt009.C      MKTAYWVMVMMMVVGFTVGGHVHRSHSPTSRSHGDDSIHDKTIHQHLFARLPLENNDDHRSVDLPAGTSAGDMKPQRQRR--LCCIF----APILWFCC------ 93
Mus005.C      MKTAYWMMVMMMVVGFTVGGHVHQSHSPTSRSHGDDSIHDKTIHQHLFARLPLENNDDHRSVDLPAGTSAGDMKPQRQRR--LCCIF----APILWFCCFG---- 95
Mil013.C      MQTAYWLMVMMMVWITAPL----------SEGGKPNDVIRGLVPDDLTPQLILRSLISRRRSDKDV-------------REGYKCVWKT-CMPALWRRHDLKGKD 81
Mil015.C      MQTAYWLMVMMMVWITAPL----------SEGGKPNDVIRGLVPDDLTPQLILRSLISRRRSDKDV-------------RADQTCIWKTWCPPSLWRRHDRKGKD 82
Mus007.C      MQTAYLVMVMMMVWYTTPV----------SECGKLNNVVRGFVPKDWTPMLPWRRLVSHTS---SKY------------PGVTFCPWKV-CPPAPRR-------- 71
Cpt010.C      MQTAYLMMVMMMVWSIGPL----------SEGDKLNDVIRGLVPKDWIPMLALRRSVSHRDTASSSP------------EGVDICIWKV-CPPTPWRRLEGQ--- 79
Mil014.C      MQTAYLMMVMMMVWSIGPL----------SEGDKLNDVIRGLVPKDWIPMLALRRPVSHRDTDSS-S------------SGAQICIWKV-CPPSPWRRLG----- 76
D-Superfamily
Mus030.D      MPKLKMTLLVLLILPLPYLNAAGGQAVQWDRRGDGLARYLQRGGRDHEMTCYPGPPGSPWGICCRTMVCGHICCPQMSCVCA-EFSDGLGCDC--    92
Mus028.D      MLKLEMMLVVLLILPLFYFDA-GGQVVQGDRRGNGLARYLQRGDRDVR-ECNINTPGSSWGKCCMTRMCGTMCCARSGCTCVYHWRRGHGCSCPG    93
Cpt045.D      MPKLAVVLLVLLILPLSYFDAAGGQAVQWDRRGNGLARYLQRGDRDVR-ECQVDTPGSSWGKCCMTRMCGTMCCSRSVCTCVYHWRRGHGCSCPG    94
Mus027.D      MPKLAVVLLVLLILPLSCFDVAGGQAAEGDRRGNGLARYLQRGDRDVR-ECNINTPGSSWGKCCMTRMCGTMCCARSGCTCVYHWRRGHGCSCPG    94
Mus026.D      MPKLAVVLLVLLILPLSYFDAAGGQVVQGDRRGNGLARYLQRGDRDVR-ECNINTPGSSWGKCCMTRMCGTMCCARSGCTCVYHWRRGHGCSCPG    94
Mus029.D      -------------LPLSYFDAAGGQVVQGDWRSDGLARYLQRGDRDVR-ECNINTPGSSWGKCCMTRMCGTMCCARSGCTCVYHWRRGHGCSCPG    81
Mil036.D      MPKLEMMLLVLLILPLSSFSAAGEQVVQGDRRSDGLARYLQRGGRDVQ-ECQVVTPGSKWGRCCLNRVCGPMCCPASHCYCIYHRGKGHGCSC--    92
Mus031.D      MPKLEMMLLVLLILPLPYFSAAGGQVVQGDRRSDGLAHYLQRGDRDVQ-ECQVNTPGSKWGKCCLNRMCGTMCCPQSHCYCIYHRERGHGCSC--    92
Mus032.D      MPKLEMMLLVLLILPLPYFSAAGGQVVQGDRRSDGLAHYLQRGDRDVQ-ECQVNTPGSKWGKCCLNRMCGTMCCPQSHCYCVYHWRRGHGCAC--    92
E-Superfamily
Mil042.E      MTRVFITMFFLLALTEGWPRLYDKNCENGPSMPPDFTCRAKEQCGTIRKRNGQLTCTLKCKCAPTGNCLNGEVIDWDDITVKTYTCP   87
Mil043.E      MTRVFITMFFLLALTEGWPRLYDKNCENGPSMHSDFTCRAKEQCGTIRKRNGQLTCTLKCKCAPTGNCLNGEVIDWDDITVKTYTCP   87
Cpt049.E      MTRVFITMFFLLALTEGWPRMYDKNCENGPSMHPDFTCRAKEKCGTIRKRNGQLTCTLKCKCAPTGNCLNGEAIDWDDITVKTYTCP   87
Mus034.E      MTRVFITMFFLLALTEGWPRMYDKNCENGPGMHPDFTCRAKEQCGTIRKRNGQLTCTLKCKCAPTGKCLNGEAIDWDDITVKTYTCP   87
F-Superfamily
Cpt050.F      MQRGVVLLGVVAFLALWPQAAAELYDLNDPEVRAMVVDGKRLMHDCALANNYIDDPWW------------------------------- 58
Mil044.F      MQRQAVLMGVVAFLTLGPQPS-ALYNWQDENVRYVANCTQTLVKYCGKAIGYMDHPWSMIRLEAFEDVRLYKAMRNEMDRCLDKLLKTT 88
G-Superfamily
Cpt051.G      MNCLQLLLVLLLISTVTALYQDGQATQRRGRNTRKMSNLLNIQTRSCPSGCPVTCPDQDECCSGITCTYNTPGGTYLR------- 78
Mil045.G      MNCLQLLLVLLLISTITALYQDGQATQRRGRNTRKMSNLLNIQTRNCPPGCPVYCHDQDECCSGITCTYNTPGGTYYCVGCGGGG 85
G2-Superfamily
Mil046.G2      MRFFFLLLTVALFLTSITGDDAERMLGMKEGGY-VREDCGSDCMPCGG---ECCCEPNSCIDGTCHHESSPN-    68
cpt053.G2      ---FYLLLTVALFLTSITGVDAGRMLRMRGGGHVVRRDCGSNCVSCGS---RCCCAPNSCVDSTCQHQSTPH-    66
Mus036.G2      ---FYLLLTVALFLTSITGGDAGRMLRMRGGGHVVRRDCASNCVSCGT---GCCCSPNSCVDSTCQHHSTPH-    66
cpt054.G2      -MRYHLMLTVILLLTLFTGGDAGPRRANRLEKHFVNRDCQSGCVGCQN-PAGCCCGELVCVNGNHCEPRSLWF    71
Mus037.G2      -MRCHLLLTVILLLTLFTGGDAGPRRANRLEKHFVNRDCQSGCVGCQN-PAGCCCGELVCVNGNHCEPRSLWF    71
Mus038.G2      MRFFYLLLTVALFLTSITGGDARLMQGMEATRPFARRDCQRGCHGCTNAPNGCCCGNLACQNEKRCQPIGR--    71
cpt052.G2      ---FHLLLTVALFLTSITGGDARLMQGMEATRPFARRDCQRGCHGCTNAPNGCCCGNLACQNEQRCEPKGG--    68
Mus035.G2      MRFFYLLLTVALFLTSITGGDARLMQGMEATRPFARRDCQRGCHGCTNAPNGCCCGNLVCQNQQRCVPKGG--    71
H-Superfamily
Cpt059.H      MSTTGRLLLLCLALGLVFESLGTPVADDVDADRDLDLDGQGLYILKSLESAEVRCGKGRCRFDCCRTIEGVEKCRELD-----------------   78
Cpt058.H      MNTSSQLLLLCLSLGLLFQCLGHPIAGGGDAARDVVPDKTSSLQSEE---RGEVP--------SAEKIRQQKKCTGVDC----------------   68
Cpt060.H      MRTSGRLLLLCLAVGLLLESQAHPIADAGDATRNVGSDGTSVELSKMIETGGQHS--------LAEKG--QRKSSDNDRYSADPSTVPDHDIPSP   85
I1-Superfamily
Cpt061.I1      MMKLLMTFLLLLMILPLCQPSGRRQLPVPDYTLEIKLDPTVAPQCYRTPCETRSQCCERHFCSAEFMCIPNADKIFRYRK 80
Mus040.I1      MMKLLMTFLLLLMILPLCQPSGRRLLPVPDYTVVIDLEPTEAPECFRTPCASRSNCCERQFCSAEHKCIPDSDKIHDYGK 80
I2-Superfamily
Cpt064.I2      -MFGHKSVNYLLLSIMALDMVATVICSCSGS-----ISSEKCESPAEKT--CTCFGCCEL-ETKADQCMTLGMCTALRSA---NNGRRAIQMTKRFHRMLRGLAD    93
Mil053.I2      -MFGHTSVSFLLLSIMTLVAAQTQLRSCSQD-----ISSEGCFDVNETCSSCAYHYCCIFQNNRPPACMTDYMCTYHQ-N---YGRRRFTQMQERFLPMLRRLAD    95
Mus042.I2      -MFRFTSVGCFLLVIVLLNLVVLTD-ACHDE-GNPCTSDNGCMCT--RCCSGVCSFH----CPS-NGKR------RRREVPVKVFGQRYV--------------    74
Cpt063.I2      -MLRFTSVGCFLLVIVLLNLVVLTN-ACHHE-GNPCTSDDGCLCT--ECCSGVCSSN----CPS-NGKR------RRREVPVKVFGQRYV--------------    74
Mus043.I2      -MFRFTSVGCFLLVIVLLNLVVLTD-ACHDE-GNPCTSDDGCCGV--ECCSGVCSSN----CPS-NGKR------RRREVPVKVFGQRYV--------------    74
Mus044.I2      -MFRLTTVSCFLLVIVLLNLVVLTD-ACHTE-ETPCSDNGQCCGV--GCCNGKCSSE----CPWEPGKR------GRRHVSFKVFGQR----------------   73
Cpt066.I2      -MFRLTTVSCFLLVIVLLNLVVLTD-ACYYDVGDPCSSNKECCIS--ECCDGICLPW----CTWPVYKR------GRRHVSFKVFGQRYV--------------    76
Mus041.I2      -MFRLTTVSCFLLVIVLLNLVVLTD-ACYYDVGDPCSSNKECCIS--ECCDGICLPW----CTWPVYKR------GRRHVSFKVFGQRYV--------------    76
Mus045.I2      -MFRFTSVGCFLLVIVLLNLVVLTD-ACYYDVGDPCSSNKECCIS--ECCDGICLPW----CTWPVYKR------GRRHVSFKVFGQR----------------    74
```

```
Cpt062.I2      MMCRLTLFCCLLLVIVPLNMAGRFD-FLQKR-ADVCSSTACDCNHDERCCQETPSYF----CRKPDECTVSYFSC------------------------------    69
Cpt065.I2      MMCRLTSLCCLLVIVLL-NSAVCHI-PCQQG-GSWCTDVMYCCDSLDVCCGTSGSAT----CTRESECSGERITHARRALYTRFFRR------------------    80
Mil052.I2      MMCRLTSLCCLLVIVLL-NSAVDGV-PCQQG-GGKCSSDLKCCDGRDVCCGTSGSAT----CTIESECSGERITHTHRALHARFFRR------------------    80
I3-Superfamily
Cpt073.I3      MKLFVAIVLVLMLLSISTGAEIPDNHGSRSATAMRDRHQWPK-SSTNRREFWRCKSNHDCSGGLCCLEN-ECRVGVAPCY-----    78
Cpt074.I3      MKLFVAIVLVLMLLSISTGAEIPDNHGSRSAAALRDRLQWPK-SSMHRRQSWRCKNNNDCFWGHCCVEH-KCVTGPVPC------    77
Cpt075.I3      MKLFVAIVLVLMLLSISTGAEMPDNHESRSAAALRDRLQWPK-SSMHRRQSWRCKNNNDCFWGHCCVEH-KCVTGPVPC------    77
Mus047.I3      MKLLLVIVLIPMLLYLSTGAETSDNRESRNGAAQRDWFHGPFYTWFCRSRGAKCRHNGQCCGGLCCAG--RCHLTFMSCGRIHFF    83
Cpt067.I3      MKLLLVIVLIPMLLYLNTGAETSDNRESRNGAAQRDWFHGPFHAWICRSRGARCRHNGQCCVGVCCAG--RCHLTFKSC------    77
Cpt070.I3      MKLLLVIVLIPMLLYLNTGAETSDNRESRNGAAQRDWFHGPFHRPFCRSRGARCRHNGQCCGDLCCVG--RCHLTFVSCRRIHFF    83
Cpt071.I3      MKLLLVIVLIPMLLYLNTGAETSDNRESRNGAAQRDRFHGPFYARFCRSRGARCRHNGQCCGDLCCVG--RCHLTFVSCRRIHFF    83
Mil054.I3      MKLLLVIVLIPMLLYLSTGAETSDNRASRSGAAQRDRLLR--DPQFCRSLGVNCSNNGQCCGDLCCVG--TCAMPMVSCGSPVF-    80
Cpt068.I3      MKLLLVIVLIPMLLYLSTGAETSDNRASRSGAAQRDRLLR--DARFCRSRGARCSHNGQCCGGVCCAG--RCHLTFKSCGRIHFF    81
Cpt069.I3      MKLLLVIVLIPMLLYLSTGAETSDNRASRSGAAQRDRLLR--DARFCRSRGARCSHHGQCCGDLCCYG--KCVLTFVSCVGS---    78
Cpt072.I3      MKLFLAIVPILMLLSLSTGVETSDNSASRTVTAAL---FS-PWLWGCSTLGEQCSIHSDCCGDLCCSGV-QCSMTYVSCN-----    75
Mus046.I3      MKLFLAIVPILMLLSLSTGVETSDNSASRTVTAAL---FS-PWLWGCSTLGEQCSIHSDCCGDLCCSGV-QCSMTYVSCN-----    75
Cpt076.I3      MKLFLAIVPILMLLSLSTGAETSDNRASRTITL----------LSTCKGKDETCSYDDDCCGSLCCFRVYGCQLAYVPC------    69
Mus048.I3      MKLSLAIVLILILLTLSTGAETSDNRASRTITL----------LSTCKGKDETCSYDDDCCGSLCCFRVYGCQLAYVPCLP----    71
J-Superfamily
Cpt082.J      MTSVWPVTCFCLLWLMLSVQL--VTPGTAQLSGRRT----ARRDMEEGLEHCPELCREGKSDPFCFCERKRDVASSWIRRRTTSIVA   81
Mus052.J      MTSVWPVTCFCLLWLMLSVQL--VTPGAAQLSGRRT----AGRNMEEGLEHCPEICREGNSDPFCLCERKRDVASSWIRRRTTSIVA   81
Mus051.J      MTSVWPVTCFCLLWLMLSVQL--VTPGAAQLSGRRT----AGRNMEEGLEHCPEICREGNSDPFCLCERKRDVASSWIRRRTTSIV-   80
Cpt084.J      MTSVRSVTCCCLLWLMLSVQ--LVTPRFTRIKRPRWESPHHTPPEGELELYCPALCNQSHGEPHCDCRRKRDMSSSWIRRILRAM--   83
Mil057.J      MMSVRSVTCCCLLCLMLSVQLHLVTPRFTRINRRLWHSPPYTPTEEEKELYCPALCNQSHGEPYCECRRKRDVISPWIRRKLRVV--   85
Mus050.J      MTLVRSVTCCSLLWLMLSVQLQLVTPRFTRINRQQWDSPNGKPSEEEKELYCPALCSQSHGEPHCECRRKRDTISPWIRRKLRVM--   85
Cpt081.J      MTSVRSVTCCCLLWLMLSEQLQLVTPRFTRINRQQWDSPNGKPSEEEKELYCPALCNQSHGEPHCECRRKRDTISPWIRRKLRVM--   85
Cpt083.J      MMSVRSVTCCCLLWLMLSEQLQLVTPRFTRINRQQWDSPNGKPSEEEKELYCPALCNQSHGEPHCECRRKRDTISPWIRRKLRVM--   85
L-Superfamily
Cpt088.L      ------------------------------EASMLADDRAANLMALLQERLCPP-MCRS-CSN-C-    32
Mil059.L      MKLSVMFIVFLMLTMPMTDGGIIRSANNGENADALAGDRATKVLELLLKSSCPP-ACCPTC-----    60
Cpt085.L      MKLSVMFIVFLMVTIPMTDGGIIRSANNGENADVLAGDRAANILELLLKRHCPP-VCCPNC-----    60
Mus055.L      -----MFIVFLMLTMPMTDGGIIRSANNGENADALAGDRAANMLELLLKRNCPP-VCCPNC-----    55
Cpt087.L      MKLSVMFIVFLMVTIPMADSDFTPRAINGEKTNTLARLKAANLKISMVKRPCPVDTCEPGCNR-CK    65
Mus054.L      MKLSVMFIVFLMLTMPMADSDFTPRAINGEKTNALARLKAANLKISMAKRPCSADTCEPGCNR-CK    65
Mil058.L      MKLSVMFIVFLMLTMPMADSDFTPRAVNETKTHKLARFRAANSL--VVKRECPV-TCEPGCEGKCK    63
Mil060.L      MKLSVMFIVFLMLTMPMADSDFTPRAVNETKTHKLARFRAANSL--VVKRECPG-TCEPGCQGRCK    63
Cpt086.L      MKLSVMFIVFLMVTIPMADSDFTPRAINGEKTHKLARFRAANSL--VVQRECPV-TCEPGCQGRCK    63
Mus053.L      MKLSVMFIVFLMLTMPMADSDFTPRAINGEKTHKLARFRAANSL--VVKRECPA-TCEPLCQGRCK    63
M-Superfamily
Cpt094.M      MSKMGVVLFTFLVLFPLATLQLNADRPVERYAKNKQDLNPYKRTIILSALSGR-GCCRTGSCHIGRSSYAVRQKCQCC    77
Mus076.M      MPKMGVVLFTFLVLFPLATLQLNADRPVERYAKNKQDLNPYKRTIILSALSGR-GCCRTGSCHIGRSSYAVRQKCQCC    77
Cpt106.M      MLKMGVLLFTFLVLFPLATLQLDADQPVERYAENKQDLNPDERMGFILSSPGRFRCCISPLC----------DGCICC    68

Cpt098.M      MGVVLFIFLVLFPLATLHLEADQPVERQQDLNVGDTRGIMRHAISKGTSFRGCCTGSACWNVPTCECCDD           70
Mus073.M      ----LFIFLVLFPLATLHLEAGQRVERQQDLNVGDTRGIMRHAISKGTSFRGCCTGSACWNVPTCECCDD           66

Cpt104.M      MMLKLRIALYIFLALFPLTTLQEKEDDVSDVEARQL--EIAEDAILMPYLNRESRDPCSSNGCDGDHNCSTGCSCIFNQ------------   77
Mus081.M      MMLKLRIALYILLALLPLTTLQDNEDDVSDLEARQLHTEIAEDAILMPYLNRESRDPCSTNGCDGDNNCSTGCSCTWNQAQRCDFCYCSSS   91

Cpt113.M      -----VLLTICLLLFPLTALPRDGDQSVDRPAERMQDDISSELHPLFNQKRMCCGEGSSCPKYFRNSQICHCC         68
Cpt115.M      -----VLLTICLLLFPLTALPRDGDQSVDRPAERMQDDISSELHPLFNQKRMCCGEGAPCPSYFRNSQICHCC         68
Mil067.M      MSKLGVLLTICLLLFPLTALPMDGDQPADQPADRMQNDISSEQYPLFDKRQKCCGPGASCPRYFKDNFICGCC         73

Cpt112.M      -MLKMGVLLFTFLVLFPLATLQLDADRPVERYAETKQDLNPDERRPNIMLTLLRRQTSCCIPPRCGGCQCC          70
Mus074.M      VMPKMGVVLFTFLVLFPLATLQLDADRPVERYAETKQDLNPDERRPNIMLTLLRRRTSCCKPPNCEGCNCC          71
Cpt101.M      VMLKMGVLLFIFLVLFPLATLQLDADRPVERYAETKQDLNPDERRPNIMLTLLRRRTSCCKPPNCEGCNCC          71

Cpt111.M      MLKMGVLLFTFLVLFPLATLQLDADQPVERYAENKQDLNPDERMKFILHALGQRQCCDWQWCD--GACDCCA         70
Mil061.M      MSKLGVVLFIFLVLFTMATLQLDADQPVDRYAENKQNLNPGERRKATRRV--RRDCCSLSACVPPPACECCK         70
Mus071.M      MLKMGVVLFIFLVLFPPATLQLDADQPVERHLENKHDLNPDERRDLMIPI--LRGCCSMSLCTPLTMCACCA         70

Cpt093.M      MLKMRVVLFIFLVLFPLATLQLDADQPVERRVERKQDFNRYERKRIIMMAALTECCAAGMCHGGCKCCC            69
Mus070.M      MLKMRVVLFICLVLFPLATLQLDADQPVERRVERKQDFNRYERKRTIMMAALTECCAADMCRNHCVCCC            69
Mus069.M      MPKMGVVLFTFLVLFPLATLQLDADQPVERRVERKQDFNRYERKRTIMMAALTECCAADMCRNHCVCCC            69

Mus075.M      MPKMGVVLFTFLVLFPLATLQLDADRPVERYAETKQDLNPDERRPNIMLTLLRRQTSCCIPPRCGGCQCC--         70
Mus077.M      -----------------TLQLDADQPVERYVENKQDLNPDERMGFILSS--PGRFRCCISPLCDGCICCIT         52

Mus078.M      MSKVGVVLLIFLVLLSLAALQNGDDPRQQRGEK-QSPQRDNFRSTLRKYSHNIQRRCRNGTSSCITCARKGKDCMGRRGGGRMCGKCVPRGR- 91
Mil062.M      MLKVGVVFLVFLVLLSLTDSWNGDNPGRQRGEK-QSPQRNVFRSNLRKYNSYQKRRCANST-PCGECTDEGKICQVQPGGKGTCGECVPNTR- 90
Cpt092.M      MLKVGVVFLVFLVLLSLADFWNMDNPGRQRGEKKQSPQMNAFRSNLRKYSSNHQRRCANST-PCGECTNEGKICQVEPGGKGTCGECVSSGR- 91
Cpt107.M      MLKVGVVFLVFLVLLSLADFWNMDNPGRQRGEKKQSPQMNAFRSNLRKYSSNHQRRCANST-PCGECTNEGKICQVQPGGKGTCGECVPSSGR 92
Mus079.M      MLKVGVVFLVFLVLLSLADFWNVDNPGRQRGEK-QSPRRNVFRSNLRKYSSNHQRRCANST-PCSECTNEGKICQVQPGGKGTCGECVSS--- 88

Cpt110.M      MMSKLGVLLTICLLLFPLTALPQDGDQPADQPAERMQNISPFKRKHWFNRAATCCYPEDDEPCDPGCKLCC          71
Mus068.M      MMSKLGVLLTICLLLFPLTALPQDGDQPADQPAERMQNISPFKRKHWFNRAATCCYPEDDEPCDPGCKLCC          71

Cpt105.M      MMSKLGVLLTICLLLFPLTAVQLDGDQPADLPTLRAQDFAP--ERSPWFDPVR-RCCSQDC-WVCIPCCPN          67
Cpt103.M      MMSKLGVLLTICLLLFPLTAVQLDGDQPADLPALRTQDIAT--DHSPWFDPVK-RCCSRYC-YICIPCCPN          67
Mus065.M      MMSKLGVLLTICLLLFPLTAVQLDGDQPADLPALRTQDIAT--DHSPWFDPVK-RCCSRYC-YICIPCCPN          67
Mus066.M      --------------------VQLDGDQPADLPALRTQDIAT--DHSPWFDPVK-RCCSRYC-YICIPCCPN          47
Cpt114.M      -------------------------------------------TILSHSV-NSCCRDCGEDCVGCCR-          23
Mus072.M      MMSKLGVLVTICLLLFPLAAFPLDGNQPADHPAKRTQDDSSAALINTWIDPSMTCC--RDCGEDCDGCCQ-          68
Mil066.M      VMSKLGVLVTICLLLFPLTALPMDGDQPSDHPAERTQDDSPVAQISSWIDDSQFCCCTQDCTSICDDCS--          69

Cpt100.M      MMSKLGVLLTICLLLFPLTVLPLDGVQGRHHLTVHPLRRLTARSSQPQHRAWDEEKKCCTGQCHICWPCCG          71
Mus067.M      MMSKLGVLLTICLLLFPLTVLPLDGVQGRHHLTVHPLRRLTARSSQPQHRAWDEEKKCCTGQCHICWPCCG          71

Mil065.M      VMSKLGVLLTICLLLFPLTALPMDGDQSVDRLAERMQDDISSEQHPLF-NQKRRCCGE-GSSCPKYFRDSQICGCC      74
Cpt102.M      MMSKLGVLVTICLLLFPLAAFPLDGNQPADHPAKRTQDDSSAALINTWIDHSHSCCRDCGEDCVG-----------      65

Mil068.M      VMSKLGVLLTICLLLFPLTALPMDGDQSVDRLAERMQDDISSEQHPLFNQKRRCCTGKKGSCSGKACKNLKCC        73
Mil070.M      ------------------------DQPADRHAERMQDDTSAAQNLWVDHDKRCCYTFCRM-----CFHLPCC        43

Cpt095.M      MLKMGVLLFTFLVLFPLATLQLDADQPVERYAENKQDLNPDERMKFILHALGQRRCCISPACNDTCYCCQDRHPRQCAGRFYTQRQRLLISCIIAPTSLV      100
Mus056.M      MPKMGVVLFTFLVLFPLATLQLDADQPVERHLENKQDLNPDERMKFILHALGQRRCCISPACNDTCYCCQDRHPRQCAGRFYTQRQRLLISCIIAPTSLV      100
```

```
Mil064.M     VMSKLGVLLTICLLLFPLTALPMDGDQPADRPAERMQDDISSEKHPLFEKRRGCCNGRGGCSSRWCRDHA--------RCCGRR     76
Mus080.M     -MPKMGVVLFTFLVLFPLATLQLDADQPVERHLENKQDLNPEERRDFIMS-----ALRGGNLAVRCHEYPQCPYASVPLCCA--     76

Cpt099.M     MSKLGVVLCIFLILFPMATLQLDGDQTADRHADERDQDLAGQYLNLKRVLKRGWVYYAHPHSNAVFSLV 69
Mus063.M     MSKLGVVLCIFLILFPMATLQLDGDQTADRHADKRDQDLAGQYQNLKRVLKRGWVYYAHPHSNAVFSLV 69
Mus064.M     MSKLGVVLCIFLILFPMATLQLDGDQTADRHADERDQDLAGQYQNLKRVLKRGWVYYAHPHSNAVFSLV 69
Cpt109.M     MSKLGVVLCIFLILFPMATLQLDGDQTADRHADERDQDLAGQYLNLKRVLKRGWVYHPHTHYNSMWSLV 69
Cpt097.M     MSKLGVVLCIFLILFPMATLQLDGDQTADRHADERDQDLAGQYLNLKRVLKRGWVYHAHPEPNSFWTLV 69
Cpt108.M     MSKLGVVLCIFLILFPMATLQLDGDQTADRHADERDQDLAGQYLNLKRVLKRGWVYHTHPEPNSWWTLV 69
Mus062.M     MSKLGVVLCIFLILFPMATLQLDGDQTADRHADKRDQDLAGQYQNLKRVLKRGWVYHTHPEPNSWWTLV 69
Mus057.M     MSKLGVVLCIFLILFPMATLQLDGDQTADRHADERDQDLAGQYQNLKRVLKRGWVYHANPEANSWWTLV 69
Cpt096.M     MSKLGVVLCIFLILFPMATLQLDGDQTADRHADERDQDLAGQYLNLKRVLKRGWVYHAHPDANSWWSLV 69
Mus058.M     MSKLGVVLCIFLILFPMATLQLDGDQTADRHADERDQDLAGQYQNLKRVLKRGWVYHAHPDANSWWSLV 69

Mil069.M     LGVVLFIFLVLFPMATLQLDGDQTADRHAGERDQDLLEQYRNLKHALKRTRNYYVVYPASPENSWWT    66
Mus059.M     LGVVLCIFLILFPMATLQLDGDQTADRHAGERDQDLVEQYRNLKHALKRTRNYYVVYSARPKNSWWS    66

Cpt091.M     MSKLGVVLCILLVLFPMATLQLDGDQVADRHVRNFPNLRRGMTRAGDPFSNTMWSL  56
Mus061.M     MSKLGVVLCIFLVLFPMATLQLDGDQVADRHVDNFPNLRRGMRRAGHPPSNIVWSL  56
Mus060.M     MSKLGVVLCIFLVLFPMATLQLDGDQVADRHVRNFPNLRRVMRRAGGSPSNSIWSL  56
Mil063.M     MSKLGVVLCILLVLFPMATLQLDGDQVADRHVRKFPNLRRVMRRAGGSPSNSIWSL  56
Cpt089.M     MSKLGVVLCILLVLFPMATLQLDGDQVADRHVRNFPNLRRVMRRAGGSPSNSIWSL  56
Cpt090.M     ------VLCILLVLFPMATLQLDGDQVADRHVRNFPNLRRVMRRAGGSPSNSIWSL  50
N-Superfamily
Cpt126.N     MMSTMGMMMLLTVLLLVPLTSLEKNLDGPIQNRDLNAGRNRFLRLLKGTTKRDCEGSRQCGGECCKNSNCICYPGMWPESPSCSTNC       87
Mus092.N     -MSTPGMKLLIL-LLLLPQEASGDGGQTIQADRHIIALGTLIRRLSREKTERTCPSGKQCGQVCCKRANCNC---------------       70
Cpt127.N     -MSTLRMMLLIL-LLLLPLATFDGDGRRIRGLRSAKLLRGI-E--PWCVGEKPCPSNRWCCKKCCPT-NCNCGWRSSDRMEREPAEC       81
Cpt125.N     -MSTLGMMPFIL-LLLLPLAAFDGDGRRIPGLRSAKLLRSVPT--PVCVRQTPCPSNKWCCESCCKKPDCPCVVVETFGYRENVCAC       83
Mil077.N     -MSTLRMMLLIL-LLLLPLAASDVYGRKIRGLRSAKLLRST----PSCVQTTPCPSNTWCCENCCERSNCWCGNGGSPGYPNLVCVC       81
O1-Superfamily
Cpt149.O1     -------------------TTTDDSTGRQEYPTKRWRAKMLNSRVFRWTEECTPPGGSCSLWYNECCSNFCILRNSGP-TC-   61
Mus107.O1     ---------------------TDDSTGRQEYPTKRWRAKMLNSRVFRWTEECTPPGGSCSLWYNECCSNFCILRNSGP-TC-   59
Cpt150.O1     ---------------ACQLITTHDSRGRQEYRAANARTKMQNYKIFRLTKGCVAPGGRCTLRHINCCSKVCKLKKNGNPVCV   67
Mus112.O1     MKLTYALIVAMLFLTACQLITTHDSRGRQEYRAANARTKMQNYKIFRLTKGCVAPGGRCTLRHINCCSKVCKLKKNGNPVCV   82

Mus114.O1     ------------------LITTD-DSRDTRRSPGLRAMTRMNSKLPRLLTRECNAQGVLCEEN-SECCSGDCTTNEVLEYC----------------    61
Cpt145.O1     MKLTCVLIVAVLFLTACQLITTD-DSSDLQEFPRRKMIDRLLDTKAAQ--ERCLGGSSPCTAFSGNCCNYCA-----FFWCT---------------    74
Mus101.O1     MKLTCVLIVAVLFLTACQLITTD-DSSDLQEFPRRKMIDRLLDTKAAQ--ERCLGGSSPCTAFSGNCCNYCA-----FFWCT---------------    74
Cpt153.O1     MKLTCVLIVAVLFLTACQLITTD-DSSDLHEFSGRKMIERLLKTKDVEAKQYCAPAEAWCNVINDKCCNYCK-----FLRCA---------------    76
Cpt154.O1     ------------FLTACQLIT-ADYSRDKQEDPVVRSSDEVQRSEDPKLAKRCSGFGVDCDPGNHNCCSGECFGFPNFGLCT---------------    69
Mil081.O1     MKLTCVLIVAVLFLTACQLITAANYARDEQEYPAVRSSDVMQDSEDLTLTKKCTDDSQFCNPSNHDCCSGKCIDEGDNGICAIVPENS---------    88
Mil085.O1     MKLTCVLIVAVLFLTACQLITAANYARDEQEYPAVRSSDVMQDSEDLTLTKKCTDSGQFCDPTDHDCCSGNCIDEGGNGVCAFVREDAPKLY-----    92
Mus094.O1     -----------------QLSTAASYARDKQEYPAVRWSDEMQDADDLTLAKKCTDDSQFCNPSSHNCCSGTCIDEGGSGVCAIVP------------    68
Cpt152.O1     MKLTCVLIVAVLFLTACQLITAASYARDKQEYPAVRSSDEMQDSEDLTLTKKCTDDSQFCDPTNHNCCSGDCIDEGGRGVCAIVPQHV---------    88
Mus105.O1     MKLTCVLIVAVLFLTACQLITAASYARDKPEYPAVRSSDEMQDSEDLTLTKKCTDDSQFCDPTDHNCCSGECINEGGRGVCAIVPVHV---------    88
Cpt129.O1     MKLTCVLIVAVLFLTACHLITTD-DSTGKQRYQAWKLRSKMQNSVLSRLSKRCDEEGTGCSSD-SECCSGRCTPEGLFEFCE---------------    80
Mus099.O1     MKLTCVLIVAVLFLTACHLITTD-DSTGKQRYRAWKLRSKMQNSVLSRLSKRCDEEGTGCSSD-SECCSGRCTPEGLFEFCE---------------    80
Cpt144.O1     MKLSCVLIVAMLPLMACHLITAD-EYREKQGYSAVRTSDKIQESEDSKLTERCMDDGDVCEVGNHDCCSGSCIGNEVDGFCAIGLEYAEGVSR----    92
Mus110.O1     MKLSCVLIVAMLPLMACRLITAD-DYREKQGYSAVRTSDKIQESEDSKLTERCTGDGDVCEVGNHDCCSGSCIGDAGDGFCAISVEYSEGVSR----    92
Mil079.O1     MKLLCVLIVAMLPLMACHLIIAD-DSSEKQGYLTVRTSDKIQESEDSKLTERCFNDGDDCEIGD-DCCSGSCVFDEYFSYCDASDPYYDDYDEYYYE    95
Mil080.O1     MKLLCVLIVAMLPLMACHLIIAD-DSSEKQGYLTVRTSDKIQESEDSKLTKRCLDDGDDCQVGD-DCCSGSCVFDEGDSFCEISYENYGGVSR----    91

Mil093.O1     ---TCVVIVAVLFLTACQLITAE-DSRGTQKHRTLRSTVRRSKSELTTRCRPSGSNCGNISI-CC-G-RCVN----RRCT--   69
Mil090.O1     MKLTCVVIVAVLLLTACQLITAD-DSRGTQKHRALRSETKLSM--STR-CKSKGAKCSRLMYDCCSG-SCSGYT--GRCG--   73
Mus097.O1     MKLTCVVIVAVLLLTACQLITAD-DSRGTQKHRALRSDTKLSM--STR-CKGKGAKCSRLMYDCCTG-SCNR----GKCG--   71
Mil095.O1     MKLTCVVIVAVLLLTACQLITAD-DSRGTQKHRALRSETKLSM--STR-CKGKGASCLRTAYDCCTG-SCNR----GRCG--   71
Cpt135.O1     MKLTCVVIVAVLLLTACQLITAD-DSRGTQKHRALRSDTKLSM--STR-CKGKGASCHRTSYDCCTG-SCNR----GKCG--   71
Cpt141.O1     MKLTCVVIVAVLLLTACQLITAD-DSRGTQKHRALRSTTKLSM--STR-CKGKGASCHRTSYDCCTG-SCRS----GKCG--   71
Mil092.O1     MKLTCVVIVAVLLLTACQLITAD-DSRGTQKHRALRSTTKLST--LTRTCTSPGGLC-GFDNNCCST-TCNVPR--NSC---   72
Cpt151.O1     MKLTCVVIVAVLLLTACQLITAD-DSRGTQKHRALGSKTKLSM--LTLRCASYGKPC-GIYNDCCNT--CDPAR--KTCT--   72
Mil089.O1     MKLTCVVIVAVLLLTACQLITAD-DSRGTQKHRALRSKTKLSM--LTLRCASYGKPC-GIDNDCCNA--CDPAR--NICT--   72

Mil078.O1     MKLTCALIITLLFLSI----TA-GDSRGKHRYNALKSMSREAN-STERECREKGQGCTNTAL-CCPGLECEGQSQGGLCVDN   75
Mil084.O1     MKLTCVLIIAMLFLTAYQLATAASYAKGKQKHRALRPADKHLR--LTKRCNDRGGGCSQHP-HCCGG-TCNKLI--GVCL--   74
Cpt138.O1     MKLTCVLIIAVLFLAACQPVTTDTFSRGKEKRRALRSTDDNSQ--LTRVCTHDGGACNNAV-HCC-G-FCNSIS--HTC---   72

Mil091.O1     ----------VLFLTACQLITPADYSRGKQEHRAVRLRDKMLRVGEFKGCAHQNQQCRP--RPCCYGLTCHVTYIP-VCI--   67
Mil094.O1     ----------VLFLTACQLITPADYSRGKQEHRAVRLRDKMQRVGEYKGCARSHQRCHP--RPCCSG-ECYMTYIP-VCL--   66
Cpt143.O1     -KLTCVLIIAVLFLTASQLIT-ADFSRDKRVHHAVRLRDIMRNFRGTRACAEFGHSCIS--ATCCPGVTCVEIDEP-VCLWD   77
Mus098.O1     -KLTCVLIIAVLFLTASQLIT-ADFSRDKRVHHAVRLRDIMRNFRGTRACAEFGHSCIS--ATCCPGVTCVEIDEP-VCLWD   77
Cpt146.O1     -KLTCVLIIAVLFLTASQLIT-ADFSRDKRVHRAERLRDLMQNFRGTRSCAELGEVCSS--TACCPDLECVEAYSP-ICLWE   77
Mus104.O1     -KLTCVLIIAVLFLTASQLIT-ADFSRDKRVHRAERLRDLMQNFRGTRSCAELGEVCSS--TACCPDLECVEAYSP-ICLWE   77
Cpt131.O1     ----------VLFLS----IT-ADDSRGKQRYSALKSIAGMLKSKTVRECRQQSEGCTNSSPPCCPGLSCRGQSQGGVC---   64
Mus109.O1     MKLTCALIITVLFLS----IT-ADDSRGKQRYSALKSIAGMLKSKTVRECRQQSEGCTNSSPPCCPGLSCRGQSQGGVC---   74
Cpt128.O1     MKLTCALIITVLFLS----IT-ADDSRGKQGYRALKSIAGMLNSKTVRECREQSQGCTNTSPPCCSGLRCSGQSQGGVCISN   77
Mus100.O1     MKLTCALIITVLFLS----IT-ADDSRDKQGYRALKSIAGMLNSKTVRECREQSQGCTNTSPPCCSGLRCSGQSQGGVCISN   77
Cpt134.O1     MKLTCVLIVAVLFLTACQLIT-ADYSRDKQEYRAVRSRDGRRNSKVPRTCAVYHESCAS--KKCCGRMECVGIQGAGLC---   76
Cpt133.O1     MKLMCVLIVTVLFLTACQLIT-ADYSRDKQEYRAVRSRDGRRNSKVPRTCAEYHESCAS--ITCCANLECIGVQGAGVC---   76
Mus106.O1     MKLMCVLIVTVLFLTACQLIT-ADYSKDKQEYRAVRSRDGRRNSKVPRTCAEYHESCAS--ITCCANLECIGVQGAGVC---   76
Mil082.O1     -------------------------------------------RECREKGQGCTNT-ALCCPGLECEGQSQGGLCVDN   34
Cpt139.O1     ------------------------------------GMRNSKVSRECREYRQGCAY--TPCCPGLKCRGTNAGGMCV--   39
Mus115.O1     MKLTSVLIVAVLFLTACQLIT-ADYSRDKQEYLAARFRDGMRNSKVSRECREYRQGCAY--TPCCPGLKCRGTNAGGMC---   76
Cpt136.O1     MKLTCVLIVAVLFLTACQLIT-ADYSRDKQEYLAARFRDGMWNSKVFWECREYRQGCTST-APCCPGLYCRGTHGGGMCV--   78
Mus113.O1     MRLTCVLIVAVLSLTACQLIT-ADYSRDKQEYLAARFRDGMRNSKVSRECREYRQGCTST-APCCPGLYCRGTHGGGMCV--   78

Mus119.O1     MKLTCVLIVVMLFLTVCPLITADYSRNKQEHPAMRLKDKMQNLKRGKWTRDCKPRRFSCAEEERCCPGLRCMTSKAGAICTT-   82
Cpt155.O1     -----------LFLTVCPL-TADYSRDKQEHPEMRLKDKMQYLKRGKWTRDCKPQSYSCAEEEPCCPGLRCMTSGAGAICTT-   70
Mus118.O1     MKLTCVVIVVMLFLTVCPLITADYSRDKQEHPAMRLKDKMQYLRRGKWTRDCKPQSFSCAGEEPCCAGLRCMTSGTGAICVT-   82
Mil083.O1     MKLTCVLIVIMLFLTVCPLITADYSRNKQEHPAMRLKDKIRYLRRGKWTRDCKPEGYSCAGEEPCCEGLRCMTSGGGAICVTQ  83
Mil086.O1     ---------------------------KQEHPAMRLKDKIRYLRRGKWTRDCKPEGYSCAGEEPCCEGLRCMTSGGGAICVTQ  56

Cpt137.O1     MKLTCMMIVAVLFLTAWTFVTADDSENGLQHRFSKALHEMNNRKASTS-DRDRKCTENGSFCLL---QDPCCGPGLCLFVCV   78
Mus108.O1     MKLTCMMIVAVLFLTAWVFITADDSRNGLENLPRMARHEIKNPEASKLNNRDDDCVAGGQGCGFPKIGGPCC-SGWCFIVCT   81
Cpt132.O1     MKLTCMMIVAVLFLTTWVFITADDSRNGLENLPRMARHEIKNPEASKLNNRDDDCVAGGQGCGFPKIGGPCC-SGWCFIVCT   81
Mus116.O1     MKLTCMMIVAVLFLTAFISITADDSRNGLENLVRKARHEMKNPKASVLNKRR-DCKANGSFCMY---YAECC-NKSCIFFC-   76
Cpt130.O1     MKLTCMMIVAVLFLTAFISITADDSRNGLENLVRMARHEMKNPKASVLNKRG-DCKANGSFCMY---YAECC-NKSCIFFC-   76
```

```
Mil088.O1   -----------------------SR---------GTQKHRSLRSTTKVSKSTSCMKAGSYCVAT--TRI-CCGYCAYFGKICIDYPKN-    53
Cpt148.O1   KLTCMMIVAVLFLTTWTFVTADDSRYGLKNLFPKARHEMKNPEASKLNKRD-GCYNAGTFCGIR--PGLCCSEFCFLW---CITFVDSG    83
Mus120.O1   -----------------------SRNGLENLPRMARHEIKNPEASKLNNRDDDCVAGGQGCGFPKIGGPCCSGWCFIV---CT------    57

Mus103.O1   MKLTCMMIVAVLFLTAWTFVTADDSINGLENRDIWGEPLSKARDKMN-PEASKLNK-RCLPPGEYCE-SGL---ECCSGICFILCL-  80
Mus117.O1   ---------AVLFLTAWTFITADDSINGLENRDIWGEPLSKARDKMN-PEASKLNK-RCLPAGEYCDLSGL---ECCSGICFILCL-  72
Mus111.O1   MKLTYILIVAVLFLTAWTFIMADGSINGPDTAGGWRKLFLKARDEMRNPAASKLNERECHEENEFCGIPFIKNGLCCSQLCIFVCL-  86
Mus096.O1   ---------AVLFLTAWTSVTADGSINRPDIAEGWQKFFSKARDEMKNRAASELNK-RCAGIGSFCGLPGLV--DCCSGRCFIVCLP  75

Cpt147.O1   ------------FLTAWTFIMADGSINGPDTAGGWRKLFLKARDEMRNPAASKLNERECHEENEFCGIPFIKNGLCCSQLCIFVCL-  74
Cpt140.O1   MKLTCMMIVAVLFLTAWTSVTADGSINRPDIAEGWQKFFSKARDEMKNRAASELNKR-CAGIGSFCGLPGL--VDCCSGRCFIVCLP  84
Cpt142.O1   MKLTCMMIVAVLFLTAWTFVTADDSINGLENRDIWGEPLSKARDKMNP-EASKLNKR-CLPPGEYCES--G--LECCSGICFILCL-  80

Mus095.O1   --------------------------GTQKHRALRSTTKLSMST-RCKGKGAKCSRLMYDCCTGSCRSG-KCG----    45
Mus093.O1   MKLTCVVIVAVLLLTACQLITADDSRGTQKHRALGSKTKLSMLTLRCASYGKPCGIY-NDCC-NTCDPARKTCT---    72
Mil087.O1   MKLTCVVIVAVLLLTACQLITADDSRGTQKHRALRSTTKLSRST-LCMSPGSPCASS-KSCC-GVCSSTQRCKKKYG    74
O2-Superfamily
Cpt167.O2   MEKLTILLLVAAVLMLTQVVIQGVGEKHENAKINFYRARKLAGNKQARVECTGWWASCGSSNYPPCCFPWECHA-YCTIPYP------ 81
Mus125.O2   ----------------------------ENAKINFYRARKLAGNKQARTECARFGAYCGSRNYPPCCSWWECEV-YCAIPGPT----- 54
Mil103.O2   MEKLTILLLVAALLMSTQAFIQGGGERRKKAVNKC--GSKPLPECWWDNDCKGWSNYCGHRH-EECCSAD-CE-YYCQLW-------- 75
Cpt176.O2   MEKLTIVILLAAVLVLAQALIKGGGEKRQKEKINFLSKRKTTAESWWEGECSGWSVYCTWDS--ECCSGE-CTRSYCELW-------- 77
Cpt171.O2   MEKLIILLLLAAVLMSTQALIQGGGEKRPMEKIRFFSKRKSNAEMWWDGECLGWSNYCRGAY--DCCSRN-CK-TYCQLW-------- 76
Mus124.O2   MEKLIILLLVAAVLMSTQALIQGGGEKRPMEKIRFFSKRKSNAEMWWDGECLGWSNYCRGAY--DCCSRN-CK-TYCQLW-------- 76
Mus121.O2   MEKLTILLLVGAVLLSTQLLA---QENPQQAKIKSFKAAMSSKN-KEMRECKKRLDPCITGQ--ECCPPMYCFDRGC----------- 71
Cpt161.O2   MEKLTILLLVGAVLLSTQLLA---QENPQQAKIKSFKAAMSSKN-KEMRECKKRLDPCITGQ--ECC--------------------- 61
Mus122.O2   MAKLTVLLLVAAVLLSTQLLVEGDGETPQRAK--FFTARKFSAV-N-KKDCDERWTICNNDG--ECCFPYSCENSNCQ---------- 72
Mus123.O2   MAKLTILLLVAAVLLSTQLLVEGDGETPQRAK--FFTARKFSAV-N-KKDCDERWTICNNDG--ECCFPYSCENSNCQ---------- 72
Cpt170.O2   MAKLTILLLVAAVLLSTQFLVEGDGETPQRAK--FFTARKFSGV-N-KKDCDERWTICNNDA--ECCIPYSCENSNCQ---------- 72
Cpt168.O2   MAKLTILLLVAAVLLSTQFLVEGDGETPQRAK--FFTARKFSGV-N-KKTCDERWTICNNDA--ECCFPYSCENSNCQ---------- 72
Cpt177.O2   MAKLTILLLVAAVLLSTQLLVEGDGETPQRAK--FFTARKFSGV-N-KKTCDERWTICNNDA--ECCFPYSCENSNCQ---------- 72
Cpt173.O2   MEKLTILLLVAAVLLSTQVLVRGGGENPQKAKINFFTKRMLSRK-N-AEICNPPEYGCMFDR--ECCSKF-CGFDSCY---------- 73
Mil098.O2   MEKLTILLLAAAVLLSTQVLVRGGRENPQKAKINFFNAKMFSGN-K-TKRCGEKHDGCDDDS--KCCSDN-CYKGVC----------- 72
Cpt174.O2   MEKLTILLLAAAVLLSTQVLVRGGSENPQKAKINFFKARMFSGN-K-AKRCAEKYQSCADDS--VCCSNN-CYQSVC----------- 72
Cpt175.O2   MEKLTILLLAAAVLLSTQVLVRGGSENPQKAKINFFKARMFSGN-K-AKRCGELYDGCDDDS--ECCSEN-C---------------- 67
Cpt178.O2   MEKLTILLLAAAVLLSTQVLVRGGSENPQKAKINFFKARMFSGN-K-AKSCAELYDGCSDDY--ECCSKS-CYKGIC----------- 72
Mil102.O2   -EKLTILVLVAAVLLLTQVMVQVDGGKPLRARITQ--------ALTRPRRCKSNGQPCEVTQ--ECCSNLYC---------------- 61
Mil097.O2   MEKLTVLILVATVLLTIQVLGQSDRDKHLKRRPKQYATKRLSALMRGTRDCTPQNVRCEEKS--ECCPNLECKCTSAPDCNF-YKCRT 85
Mil099.O2   --------------------------------------KRLSALMRGTRDCTPQNVRCEEKS--ECCPNLECKCTSAPDCNF-YKCRT 47
Mus131.O2   MEKLTVLILVATVLLTIQVLAQSDGDKHLKRRPKQYATKRLSTLMKGHRQCIPQNMDCEEDH--QCCPNLECKCISAPDCNV-PKC-- 83
Cpt164.O2   -----------------------------------------SLVFFRHRQCTPQNVQCEEDD--ECCSNLECKCSTVPDCNF-PKCRP 44
Cpt158.O2   MEKLTVLILVATVLLMIQVLAQSDGDKHLKRRPKQYATKRLSALMRGHRQCTPQNVQCEEDD--ECCSNLECKCSTVPDCNF-PKCRP 85
Mus128.O2   MEKLTVLILVATVLLMIQVLAQSDGDKHLKRRPKQYATKRFSALMRGHRQCTPQNVKCEEDD--ECCPDLECKCFTIPDCNF-PKCRP 85
Mil096.O2   MEKLTVLILVATVLLTIQVLGQSDRDKHLKRRPKQYATKRLSALMKGHRQCTGAGFECEETP--ECCPNLTCKCSGSPLCTR-YRCKT 85
Mil101.O2   --------------------------------------RTSFVFFRHRQCTGAGFECEETP--ECCPNLTCKCSGSPLCTR-YRCKT 46
Cpt159.O2   MEKLTVLILVATVLLTIHVLVQSDGDKHLKRRPKQYATKRLSALMRGHRQCTQQGYGCDETE--ECCSNLSCKCTGSPLCTL-YKCQ- 84
Mus132.O2   -----------------------------KRRPKQYATKRLSALMRGHRQCTQQGYGCDETE--ECCSNLSCKCTGSPLCTL-YKCQ- 55
Cpt156.O2   ------------------------GDKHLKRRPKQYATKRLSALMRGHRQCTQQGYGCDETE--ECCSNLSCKCSGSPLCTR-HKCKP 61
Cpt166.O2   MEKLTVLILVATVLLTIHVLVQSDGDKHLKRRPKQYATKRLSALMRGHRQCTQQGYGCDETE--ECCSNLSCKCSGSPLCTR-HKCKP 85
Cpt157.O2   MEKLTVLILVATVLLTIHVLVQSDGDKHLKRRPKQYATKRLSALMRGHRQCTQQGYGCDETE--ECCSNLSCKCSGSPLCTS-SYCRP 85
Mus129.O2   -----------------------------KRRPKQYATKRLSALMRGHRQCTQQGYGCDETE--ECCSNLSCKCSGSPLCTS-SYCRP 56
Cpt163.O2   ---------------------QSDGDKHLKRRPKQYATKHLSALMRGPRQCTPQGNFCEEDE--ECCPNLECKCSGSPLCTR-YKCK- 63
Cpt169.O2   ---------------------QSDGDKHLKRRPKQYATKHLSALMRGPRQCIPQGNMCGEED--ECCQNLECKCSHSPLCTR-YKCKP 64
Mus130.O2   MEKLTVLILVATVLLTIQVLAQSDGDKHLKRRPKQYATKRLSALMRGPRQCIPQGNMCGEED--ECCQNLECKCSHSPLCTR-YKC-- 83
Mus127.O2   MEKLTVLILVATVLLTIQFLDQSDGEKHLERRVKQHATKRLSALMRGPSNCTGQGLACEKAG--DCCPPFVCKCITSTDCHSGLKC-- 84
Cpt160.O2   MEKLTVLILVATVLLTIQALAQSDGEKPLKGRAKQHAAKSFSALMRGPRECTENNKACEEDG--DCCSN-ICRCNTRPDCTSGYKCR- 84
Cpt172.O2   ------------------------------------------------RECTENNKACEEDG--DCCSN-ICRCNTRPDCTSGYKCR- 36

Cpt180.O2   ------------AHERAEAEEPQHHRAKRQDDMAEVNDYPLDDVDMMQRIFRTPLKRQWCRSGMSYNPVLGTCTLSLAALRGRG 72
Mil104.O2   LVLVAAVLLSTQVMVQGDGDQPAYRNAVPRDDNTGGASRKFLN---VPRE-SECPWRPWCG----------------------- 57

Cpt179.O2   MEKLIALMVVAAVLVLIEVKVQGNSPKDVHKRLRDSFVQRVRRCYEASEPCTENSECCSPYVCRCTSGSVCNAKC  75
Mus126.O2   MEKLIALMVVAAVLVLIEVKVQGNSPKDVHKRLRDSFVQRVRRCYEASEPCTENSECCSPYVCRCTSGSVCNAKC  75

Mus133.O2   MEKLTMLILLATALLSIQVMAVGDGEKPVMGRAAAHRLFGLMRGRTCKSEDYHCQRDSECCDPLDCRCTVPSNCAPINIRCK   82
Cpt165.O2   MEKLTMLILLATALLSIQVMAVGDGEKPVMGRAAAHRLFGLMRGRTCKTEDYLCQRDSECCEPLDCMCTVPSNCASTNIKCK   82
Cpt162.O2   MEKLTMLILLATALLSIQVMAVGDGEKPVMGRAAAHRLFGLMRGRTCKTEDYLCQRDSECCEPLDCMCTVPSNCAPINIRCK   82

Mil100.O2   MEKLTILLLVAAVLMLTQVVIQGVGEKHENTKMNFYKARKLAGNKHSRSSDEECVGLSGYCGPWNNPPCCSWWECEVYCAVPGPSFK
O3-Superfamily
Cpt187.O3   MSGSGVALLAFLLLLSLMI-NLQGGGEGQTMHQNKHRQTVRKLLDLGRTQTRNACEFDTSLGDDCTGTQICCNPPGTASGWCTEARDCAHGRR    92
Cpt181.O3   --------------------NLQGGGEGQTMHQNKHRQTVRKLLDLGRTQTRNACEFDTSLGDDCTGTQICCNPPGTASGWCTEARDCAHGRR    73
Cpt182.O3   MSGSGVALLGFLLLLSLAT-NLQGGGEGQTMHQNKHRQTVRKLLDLGRTQTRNACEFDTSLGDDCTGTQICCNPPGTASGWCTEARDCAHGRR    92
Cpt183.O3   MSGLPILLLTIVLL-VTMGTGRQGKEKELMQRQKRLGKWPYMIRTLQRR-SCEECAYDS-DCNSCTGKPKCCIDVETWEGECYDSC-------    83
Mus135.O3   MSGLPILLLTLVLL-VTMGTGRQGKEKELMQRQKRLGKWPYMLRTLQRR-SCEECAYDS-DCNSCTGNPKCCIDVETWEGECYDSCRSK----    86
Cpt185.O3   --GSGGMLP-ALLLLVALATSQFGAQGRRM----------ERRRMLQRRKDCERNH-----HEECPSGQECCTFPNAERGTCKVSC-------    68
Mil105.O3   MSGSGVMLLALLLLLVALATSQFDAQGRRM----------ERRRKLQRRTDCERNH-----HEECPAGQECCTFPNAERGTCKVSC-------    71
Cpt186.O3   MSGLGIMVLTLLLL-VFMATSHQDAGEKQATQRDAIN--VRRRRSITRRVDE-ECN-----EYCDDRNKECCGRTNGHPR-CANVCFG-----    78
Mus134.O3   MSGLGIMVLTLLLL-VFMATSHQDAGEKQATQRDAIN--VRRRRSITRRVDE-ECN-----EYCDDRNKECCGRTNGHPR-CANVCFG-----    78
Cpt184.O3   MCGLGIMLLALLLL-VSMETSLRGRGEGHAVLRDESQ--AGRRKLALRRALQ-RLH-----KPPRGT---------KKSS-SFDVPFAAFVA-    73
Mil106.O3   MCGLGIMLLALLLL-VSMETSLHGRREGHAVLRDGSQ--AGGRKLALRRALQ-RLR-----KPPRGT---------KKSS-SFDVPFPAFVA-    73
P-Superfamily
Mil107.P    ------GSAVLVLLLLFALGNFAGVQPGQITRDVDNGQLTGNRRNLRSRWRLMSLFE---ARDSCAGSCQEDSDCDPPSCTC------------------------    73
Cpt188.P    --LSLAGSAVLMLLLLFALGNFVGVQPGQITRDVDNGQLTDNRPNLRSLWKPMRLLKPLDKRHTCNGLCVTDHDC-------------------------------    73
Cpt190.P    MHLSLAGSAVLMLLLLFALGNFVGVQPGQITRDVDNGQLTDNRPNLRSLWKPMRLLKPLDKRHTCNGLCVTDHDCHENHVGHVHSGDDHHHCHCDLFVCHHDSDVD    106
Mus137.P    --LSLAGSAVLMLLLLFALGNFVGVQPGQITRDVDNGQLTDNRPNLRSLWKPMRLLKPLDKRHTCNGLCVTDHDC-------------------------------    73
Mus136.P    MHLSLAGSAVLMLLLLFALGNFVGVQPGQITRDVDNGQLTDNRPNLRSLWKPMRLLKPLDKRHTCNGLCVTDHDCHENHVGHVHSGDDHHHCHCDLFVCHHDSDVD    106
Cpt189.P    ----------LMLLLLFALGNFVGVQPGQITRDVDNGQLTDNRPNLRSRWQPMSLFKTLDKRGWCASSCTTNGDCGSPCYCTT-----GGHC--------------    77
Mus138.P    --------------------------PGQITRDVDNGQLTDNRPNLRSRWQPMSLFKTLDKRGWCASSCTTNGDCGSPCYCTT-----GGHC--------------    61
Q-Superfamily
Mus142.Q    MSTRGTILLILLFLLPLEAFYGDGQTSRRSSKALRNIYSRVRSGCEEEPCTLVSWCCVNCCPKSDCQCQIINPGPHQTLQCNC
R-Superfamily
Mil111.R    MRASTWLSGRMVITVLPSLRVSVAISTLSGVSLVRSRLLLSTLTARPRASSRLVSPSLYSCFSRDTALALLLPMHVAFQPP 81
Cpt193.R    MRASTWLSGRMVITVLPSLRVSVAISTLSGVSLVRSRLLLSTLTARDRASSRLVSPSLYSCFSRDTALALLLPMHVAFQPP 81
Mus143.R    MRASTWLSGRMVITVLPSLRVSVAISTLSGVSLVRSRLLLSTLTARDRASSRLVSPSLYSCFSRDTALALLLPMHVAFQPP 81
S-Superfamily
Mus145.S    MMWKLGAMLFVLFLLFPLASLQQEGDVRAVKTDLKSNSYGPYQMSARDCSGTC--GQTSACTGTCNCKGHESCTCSNSG--QHSGCTCSC----    86
Cpt194.S    VMWKMGAM-FVLLLLFTLTSSQQEGDVQARKMRSKNVFHRTMAKTTRGCEGTCYGSEETNCNGSCECSSGGECYCGYY----RGSCICTCAG--    87
Mus144.S    VMWKMGAM-FVLLLLFTLTSSQQEGDVQARKMRSKNVFHRTMAKTTRGCEGTCYGSEETNCNGSCECSSGGECYCGYY----RGSCICTCAG--    87
```

```
Mil112.S       MMSKMGAM-FVLLLLLTLASSQQEGDVQARKTSLKSDFYRALRPYDRQCTFVNNCQQNGACNGDCSCGD-QICKCGYRIRPGRSGCACTCRNAK    92
T-Superfamily
Cpt203.T       MLCLPVFIILLLLASPAAPNPLERRIQSDLIRTALEDADMKTDERFL----GLIGPITSIAGKLCCTVSVSFCC--  70
Mus148.T       MLCLPVFIILLLLASPAAPNPLERRIQSDLIRTALEDADMKTDERFL----GLIGPITSIAGKLCCTVSVSFCC--  70
Cpt210.T       MLCLPVFIILLLLASPAAPNPLERRIQSDLIRTALEDADMKTEKGVLSGIMSNLGTVGNMVGGFCCTVYSGCCAEK  76
Mus147.T       MLCLPVFIILLLLASPAAPNPLERRIQSDLIRTALEDADMKTEKGVLSGIMSNLGTVGNMVGGFCCTVYSGCCAEK  76
Mus150.T       MLCLPVFIILLLLASPAAPNPLERRIQSDLIRAALEDADMKTAKGILSNIMGNLGNIGNMAGSFCCSVYSGCCPEK  76
Cpt206.T       MLCLPVFIILLLLASPAAPNPLERRIQSDLIRTALEDADMKTEKGILGSIMGNLGNIGNVAGSVCCSVYSGCCPEK  76
Mus151.T       MLCLPVFIILLLLASPAAPNPLERRIQSDLIRTALEDADMKTEKGILGSIMGNLGNIGNMAGSFCCSVYSGCCPEK  76

Cpt208.T       MLCLPVFIILLLLASPAAPNPFETKLQSDLTRADA---DMKTDKNLDAVRKAICCNVYPGTDCCMG------- 63
Mus152.T       MLCLPIFLILLLSLSSAAG---FPELQRYLTQHSPKDSGMRTNHL---------FLKKVGDDCCVGNVGTCC- 60
Mil119.T       MLGLPIYIILLLLVSPAATLAVESELQRDLTHESPKDFGMRTEHL---------RVLNVGDDCCVDNVGTCCT 64
Cpt204.T       MLGLPIYIILLLLVSPAATLAVESELQRDLTHESPKDFGMRAE--------------PVGDDCCVDNVGTCCT 59
Mus157.T       MLGLPIYIILLLLVSPAATLAVESELQRDLTHESPKDFGMRAE--------------PVGDDCCVDNVGTCCT 59
Mus159.T       MLGLPIYIILLLLVSPAATLAVESELQRDLTHESPKDFGMRAE--------------PVGDDCCVDNVGTCC- 58

Mus161.T       MRLPVFIILLLLIPFPPSVDARPKTKDDVPLASFHDNAMSPEQRD------LCCRPGNLYCCVD-    59
Mus149.T       MRCLPVLVILLLLIASTPTVDARPKTKDDVPPASFHGADNANRILRTLWNLRGCCED--KTCCFIG    64
Cpt199.T       MHCLPVLVILLLLIASTPTVDARPKTKDDVPPASFHGADNANRILRTLWNLRGCCED--KTCCFIG    64
Cpt211.T       ----PVLVILLLLIASTPTVDARPKTKDDVPPASFHGADNANRILRTLWNLRGCCED--KTCCFIG    60

Mus164.T       MKCLPVFFIVLLLIAHSSQVAEHSKLGRIKMLR-RLTAAMILEDE-GFPELCCPSDKDCCEALSN 63
Cpt205.T       ---LPVFIILLLLVSPAATLPVESELQRDLTQVSAEDFGMRTEYERRKQWGDCCNNGDCC----- 57
Mus156.T       ---LPVFIILLLLVSPAATLPVESELQRDLTQVSAEDFGMRTEYERRKQWGDCCNNGDCC----- 57
Cpt209.T       MLGLPIYIILLLLVSPAATLPVESELQRDLTQVSAEDFGMRTEYERRKQWGDCCNNGDCCS---- 61
Cpt202.T       ---LPVFIILLLLVSPAATLPVESELQRDLTQVSPKDFGMRPEHKRRMQWIGCCGRGVCC----- 57
Mus163.T       ---LPVFIILLLLVSPAAALRVESDLQRDLTQVSPKDFGMRPEHKRRMQWIGCCGRGVCC----- 57


Mus162.T       MLGLPIYIILLLLVSPAATLPD------------FGMRTELKL-KRRMQRPTCCGRGI-CCS--- 48
Mus153.T       MRCLPVFVILLLLIASTPSVDATQKTKDDMSLASFHDNAKRFLQTLRNTR-SCCPEEITCCPWG- 63
Mus160.T       MRCLPVFIILLLLIPFPPSVDARPKTKDDVPLASFHDNAERTLLRQWNKR-TCCQFYPSCCP--- 61
Cpt201.T       MCCLPVFIILLLLIPSAPSVDARAKTKHDVPLASFHDNVRRTLQTLWKTR-QCCTTQSGCCPWGK 64
Mus155.T       MCCLPVFIILLLLIPSAPSVDARAKTKHDVPLASFHDNVRRTLQTLWKTR-QCCTTQSGCCPWGK 64
Mil118.T       MCCLPVFIILLLLIPSASSVDVQPLTKDDVPLASFLANARRTLQSLWMTR-RCCPKKPYCCPGGK 64
Cpt207.T       MRCLPVFIILLLLIPSAPSLIAKPKTEDYVPLASFHGNTKRTLQILRKDIEECCPMEEHCC---- 61
Mus154.T       MRCLPVFIILLLLIPSAPSLIAKPKTEDYVPLASFHGNTKRTLQILRKDIEECCPMEEHCC---- 61
Mus158.T       -----VFIILLLLIPSAPSILARPKTEVDVPLDSLDDNAKRTLQTLWNARNECCDDRPWCC---- 56

Cpt200.T       MRCLPVFIILLLIQLAPSVDARLKIKDDAPLASSQDNPMRTRQHWCCTPLRTWCCPIVEGKLWIRPLQTVPGCEIWKADCSFCTCSWNFEWSLTTRCHLQATISLSFHLWNWIIKQLKCHRNF
U-Superfamily
Mil120.U         MNRMGFFLMLTAAVLLTSLICTEAAPADEAKMERAQQSNRDRSRNPEKRCVDCRPGYQCCGVCTGNHCTGQEIPKE
V-Superfamily
Cpt213.V       -----MMPLVLLLLLSLATHCGDGQAIQGDR------RLSARLLRGYKERGLS-IKTCGTCNGARCCGLCPCSPGEKNCSCLPFGK    74
Mus165.V       -----MMPLVLLLLLSLATHCGDGQAIQGDR------RLSARLLRGYKERGLS-IRECGTCNGARCCGLCPCSPGEKNCSCLPFGK    74
Mus166.V       MSAPGKMLLILLLLLPQETCCGDGQAIQVDGDSSAASRRPTRLLKGYKEHDRSDDNRCKLCNDVKCCGWCTCLEK-EDCRCTPMGK    85
W-Superfamily
Cpt216.W       LTVSNSVSSPRPWPCFDSVGVTCIGKTPD-------------------------GCRWTGVVAPSGRAP-------RRSNSCQLGALDGGVLRLLDQDRHH 69
Cpt215.W       MVTSVSMSSLRLWPWFDSVSVTSIIESMGAVVDGRGGALWRESPL------------------MLALQLSNSNLFPPKQLLLSAGSLVSGVLHMLDKNRHH 83
Cpt217.W       ---------------------------------------------GHRYRPVHGGCHWKDVVAPFEGELT-HAGSVTEQLLLSRGGLVGGVLRMLDQNRGH 55
Cpt223.W       MVTLRSMSFPRRWSWFGSVGVTAVDQFIGAVIGH----YG------------------------LQGKLA-DAGSAVKQLLLSVGSLVGDVLHRLDQNR-- 70
Cpt220.W       -------------------------ESMGVVVGR----MWWLAPSGRKAHPCWLHDAIVNKEHPLEGELA-CAGCATKQLLSSVWGLLCSVLHMVDQNRCH 71
Cpt221.W       MVTSVSMSAPHLWPWFGSVGVAGIGESMGVVVGR----MWWLAPSGRKAHPCWLHDAIVNKEHPLEGELA-CAGCATKQLLSSVWGLLCSVLHMVDQNRCH 96
Cpt218.W       ---SISISSPHLWPWYGSVGINGISKSMGAVMGW----TWRPL---------------------EGELTT-HAGSATKQLLS------------------- 53
Cpt214.W       -VISVSMSSPRLRPWFNSVGVTDIGKITGAVVGR----MWRRP---------------------PEGELT-HAGYGTKQL-SRL----------------- 56
Mil121.W       -VISVSMSSPRLWPWFNSVGVTDIGKITGVVVGR----MWRRP---------------------PEGELT-RAGYGTKQL-SSLGDLVNDVLRMLDRNRRH 73
Mus168.W       -VISVSMSSPRLWPWFNSVGVTDIGKITGVVVGR----MWRRP---------------------PEGELT-RAGYGTKQL-SSLGDLGNDVLRMLDRNRRH 73
Cpt222.W       -----------------SVGVSGIGEFMRAVVGR----TWWRP---------------------PEGELT-HAGSTTKQLLSSVGGLVGGVLGMFHWNRRY 58
Cpt219.W       ---SVLMFSSFLWPWFGFVGVTGIGKSMGAVVGQ----MWWCP---------------------LEEELT-HASSATKQFLSSVGGLLAGVLHLLD----- 67
Mus167.W       MLTSVSMSSSRLWPWFSFAGVTSIGKSMEAVVGR----TWWHP---------------------SGGELT-HAGSATKQLLSSEGGLVGGVVHMLDQNARH 75
Z-Superfamily
Cpt224.Z       MVKYMTSKWGVCIILPVWLVYL----------------------------------SCQ-----TQMQALPCHPVLCSGVTLHICLAE--    49
Cpt225.Z       ----MP---ALPSPLVLWFFGFFVHASLNEGQTICTGLLPGAEVGGPHVTIFLLSQYCVSHTKFTASEQINCHRMLLSDMVSITAKQGIY    83
Cerm03
Mil016.Cerm03       MHLSVMLVVLLLTMPLFSGFVHNTITGRKTVVPKDHSAEYPKIRQEPCPSGCKSCDPPGSCQES 64
Cpt011.Cerm03       MHLSVMLVVLLLTMPLFNGFVHNTITGRKTVVPKDHSAEYPKIRRQPCPASCRSCSPPGTCQAS 64
Mus008.Cerm03       MHLSVMLVVLLLTMPLFNGFVLNTITGRKTVVPKDHSAMYPNIRQEPCPAGCRSCKPPGTCQES 64
Cerm06
Cpt012.Cerm06  MTEAAIKVCEAGGSKILGDASPMATSLCAPVCTAALAKLEEVA
Pmag-02
Mus139.Pmag-02      MKAVAVFLIVSLAVAYGQFFCPESVNDPLNCIETQANSATCMQSSVDGSYSYACGYCGKKKETCFGNKAAVQDYYCQRNGIANNCGG---  87
Cpt192.Pmag-02      MKAVAVFLVVAVAVAYGQFFCPDSENDPLNCVETKGTEPACMKS-KDGSYSYACGYCGKKKESCFGNKVPVADYACQIRKIPNPCGGTAL  89
Mil108.Pmag-02      MKVVAVFLVVALAVAYGQFFCPGSKDAPLNCIETKGTSATCMRS-GDGTYSYACGYCGKKNETCFGNKVPVKDYYCQISKVANNCGGAAL  89
Cpt191.Pmag-02      MKVVAVFLVVALAVAYGQFFCPSSKDAPLNCIETMGTKATCMRS-EDGSHSYACGYCGKKKEACFGNKVPVKDYYCQINKVPNNCGGTAL  89
Mus140.Pmag-02      MKVVAVFLVVALAVAYGQFFCPSSKDAPLNCIETMGTKATCMRA-DDGSHSYACGYCGKKKEACFGNKVPVKDYYCQINNVANNCGGTAL  89
Mil109.Pmag-02      ---------------YGQFFCPDNENDPLNCIETMASGATCMKSNKDGSYSYACGYCGKKKESCFGDKVPVTNYHCQTRKIPNKCGGPVL  75
Mil110.Pmag-02      MKVVAVFLVVALAVAYGQFFCPSSKDEPLNCIETMASTATCMKSTADESLSYACGYCGKKKETCSGDKVPVTNYNCQIKKIPNPCGGPAL  90
SF-im6
Cpt195.SF-im6     MRVFPYCLAAALVVVCLSRVSEANILKSGHNETRAVDASGDDCVDSNENCAGWASTGQCEANRGYMLTNCRSF-im6
SF-mi1
Cpt197.SF-mi1       ---TGLVLVVLYLLSSPVNLQQTEDDKAFMKIMIPTLTRVLERSGSCVPKSGGGCTGTCPG---TCEGSIKGCACKTTG  73
Mus146.SF-mi1       MSKTGLVLVVLYLLSSTVNLQQTEDDKAFMKIMIPTLTRVLERSGSCVPKSGGGCTGTCPG---TCEGSIKGCACKTTG  76
Cpt196.SF-mi1       MSKTGLVLVVLYLLSSPVNLQQNEDDQAFSKIETRD----RPACYNCFPDERGHCVGTCCNASDNCRGGIRGCGCV---  72
Mil113.SF-mi1       MSKTGLVLVVLYLLSSPVNLQQNEDDQAFSKIETRD----RPECYNCFPNDDGHCVGTCCG-EDSCKGGIRGCGCL---  71
SF-mi3
Cpt198.SF-mi3       MGILTVLLPLVVVLVLTQVMVGSDQDKPLNRRRNIREAQVQRRVRDCDRQGDDCEANTECCFGLICKDEACEHVSPGPGRRAVENRAAIIRENKP  95
Mil114.SF-mi3       MGILTVLLPLVAVLVLTQVIVRSDQDKPLNRRRNIREAQALRRVRDCNIQEDACESNDDCCLGLFCKNEVCEYAS--PGRRAVENRAALIRGNKP  93
SF-mi4
Mil115.SF-mi4       MTPRMNLLLMTFVVMTVPLLLAQPNVRCETNDNGDIICKKANGQTLHNPDRSDNGTTDLRQEGPLMTALRGPWHR  75
Mil116.SF-mi4       MTPRMNLLLMTFVLMTVPLLRAHPNVRCETNDNGDIICKKANGQTLHNPDRSDNGTTDLRQEGPLMTALRGPWHR  75
SF-mi8
Mil117.SF-mi8       MMYRLTLFCCLLLVIVPLNMARKSGVLPRWNEICSPTACYCNHEERCCRETPSYFCKKADECTVSYFA
Ggeo01
Mil048.Ggeo01       MSRLFLVLLVISVVILHTDSSQGTDGSPDVGSRPLSRAATGDGFQGKLRA---RIF----RRG-ALSDKTDSEEEETGEKE-LGKRQQAV--QKRGIRRMKQWNINKKY    98
Mil047.Ggeo01       MSRLFLVLLVMSFFTFDTDSTQGHDGGTDKSSRPVARAARNRATPALFRKFQARRTFR--VHGRAVDDHTGNEEDE--EDESFDDPQHRLLKTMKNLKAFSVLNLH---    102
Mus039.Ggeo01       MSRLFLVLLAMSVFTFHTDSTQGHDGGTDKSSRPMARAARNRATPALFRKFQARRTFR--VHGRAVDDHTGDEEDE--EDESFDDPQHCLLKAVKNLKAFSVLNLH---    102
Cpt055.Ggeo01       MSRLFLLLLVMAVFTFHTDSTQGHDGGTDKSSRPMARAARHRATPALFRKFQARRTFR--VQGRAVDDHTGDEEDE--DDESFDDPQHCLLKTVKNLKAFSVLNLH---    102
```

```
Cpt056.Ggeo01      MSRLFLVLLVMSVFTFHTDSTQGHDGGTDKSSRPMARAARHRATPALFRKFQARRTFR--VQGRAVDDHTGDEEDE--DDESFDDPQHCLLKTVKNLKAFSVLNLH---    102
Cpt057.Ggeo01      MSRLFLVLLVITALTLHTDSTQGLDGDADKSSRPVARAARDHASPALLRKFRARANVRSRRVGRAVEDFPGGEEEAAEEAEEFDDGTNAMLIQKLKNHFFNLYHFH---    106
Mil049.Ggeo01      MSRLFLVLLVMSFFTFDTDSTQGHDGGTDLSSRPMARAARDHASPALLRKFRARTNVRSRRTGHAFSDDSG--------------------------------------    71
Ggeo03
Mil050.Ggeo03      MKMYLCLAILLLLASTIVDSVLVDKIQTIRKWRRRDIHCHGCNCYELRTARCTENIYCNTPPECKPRVSCNGLGGCACTRFASGRCTRTVECIPNKC    97
Mil051.Ggeo03      MKMYLCLAILLLLASTIVDSVLVDKIQTIRKWRRRDIHCHACNCFELNTARCTDKISCITPEACIPRISCPITGDCVCTRFASGCCTRAVECAPGKC    97
Divergent_MSTLGMLLL
Mus033.Divergent_MSTLGMLLL      MSTLGILLLIALLLPLTNPADNGDGQAKSRSRTLRSWRTSYTLRRLDKRACDPTSGCGTVVCKTETGPCCCKPNYECETSLSGRVACICHGDVSDCPV  98
Cpt048.Divergent_MSTLGMTLL      MSTLGMLLLIALLLPLTNPADNGDGQAKSRSRTLRSWRSSYTPRRLDKRACDPTSGCETVVCKTETGPCCCKPNYECETSLSGRVACICHGDVSDCPV  98
Mil039.Divergent_MSTLGMTLL      MSTLGMLLLIALLLPLTNPADNGDGQAKSRSRTLRSWRTSYTLRRLDKRACDPIEGCTTEVCNTETGPCCCRPNFECETSISGRIACICHGQPSDCPL  98
Mil040.Divergent_MSTLGMTLL      ----------------------------------------------DKRTCDPIEGCATEVCNTETGPCCCKPNYECETSKSGRVACICHGQPSDCPL  52
Divergent_MSKLVILAVL
Cpt047.Divergent_MSKLVILAVL      MSKLAIALLFFLLLVLVTDQHNPDDQAVRVAKRNFKMFRSLASGKRDDECNGTEACNEDGECDGCSCVDFNGEGKCSSITPP 82
Mil037.Divergent_MSKLVILAVL      MSKLTIALIFLLLLVLVTDQRNPDDQAVRLAKRNFKKFRSLSSGRRADECNGTEACDEDDDCDGCSCVDFDGEGKCASITPP 82
Mil038.Divergent_MSKLVILAVL      MSKLTIALIFLLLLVLVTDQRNPDDQAVRLAKRNFKKFRSLSSGRRADECNGTEACDEDEDCDGCGCVDFNGEGKCASVTSP 82
Divergent_MKFPLLFISL
Cpt046.Divergent_MKFPLLFISL   MKFPTFVMVLMAAVLLTSILETEASTSFRARLKAKREWEEYPCADTFADCRGQPDGTKCCDSGHCEGSVCYY
Divergent_MSTLGRVLLL
Mil041.Divergent_MSTLGRVLLL    MSTLGKVLLLLLLLLPLGNPDGDGDRQAMDRDRTASEARSAARLRLRRYMDHGRPADKRCSGVQCGEKCCDDSCECTVVGQQSNEIGCSCPV
MEALT
Cpt116.MEALT      MEALTIFRLCLLAALTTSVVVSAPLHTREQKGGCPVGGGPNPLLHCMIACMTTSTRYLCHVYCKDCSRGYDGDGHS 77
Cpt117.MEALT      MEALTIFRLCLLAALTTSVVVSAPLHTREQKGGCPVSGGPNPLLYCMMACMTTSTHYLCRHDYCKDCSRGYDGDGHS 77
METSSS
Cpt118.METSS      METSSSSSVVSAVWLRNVLAVDVVVCVFSILTGCVRILPTNSSRSFWWIHSTRPSDVTPRPTGSATLSTSTVRCVA  76
Mus082.METSS      METSSSSSVVSAVWLRNVLAVDVVVCVFSILTGCVRILPTNSSRSFWWIHSTRPSDVTPRPTGSATLSTSTVRCVA  76
Mil071.METSS      METSSSSSVVSAVWLRNVLAVDVVVCVFSILTGCVRILPTNSSRSFWWIHSTRPSDVTPRPTGSATLSTSTARCVA  76
MGTMK
Cpt119.MGTMK      TMKAALFILLVLTLGTLGVNGEDGRMMQGETPSNTYIRTVIRSARKGSDQCDHPTCGTCCLNGQRCECIKIQCD    74
Mus083.MGTMK      TMNAALFTLLVLTLGTLGVNGEDGRMMQGETPSNTYTRTVIRSAGRGSDQCDHPTCGTCCLNGQKCECTKVQCD    74
MIKSL
Mil072.MIKSL      MIKSLVILLFGCLALTASSITWRNRKPCCVNARAPCDWATCPNYPTAPCWNLPCQCRAAFRSRGRFLTPSECNGR
MKIAL
Mus084.MKIAL      MKIALIICLLSIAFMTGDGSSGDTMYSQEKAGTVSAIKRFQKKFLRRTCVNCPEEPCCYGDKCMYDPGYEPFCGN
MMLFM
Cpt121.MMLFM     MMLFMLAAVILTMASTTVNAETCANEKKVCHAYMSGVKELDICNCASTGGCTKDDDHKLTVTSSTFYICQEISEFDVCDESQAPMALTLQLNCRCASGTYLIENNTIVC 109
MMTLK
Cpt122.MMTLK      --------LFTLLLLPLATIRAEVYACTYWTSPGWAVKRCQTHADCVPYCGTHFRCRCQPGANLCPGGAGCF  64
Mus086.MMTLK      MMTLKHVLLFTLLLLPLATIRAEVYACTYWTSPGWAVKRCQTHADCVPYCGTHFRCRCQPGANLCPGGAGCF  72
MNVRM
Mil073.MNVRM      -----------------SVDLATPRHEKKADDARCRQGEYSQCPDYYVNCVAECLGHEKGMGHCTTECPLHGPPTGL 60
Mus087.MNVRM      MNVRMTLAVLVLVALTTSVDLATPRHEKKADDARCRQGEYSQCPDFYVNCVAECLGQEKGMGHCTTECPLHGPPTGL 77
MQFLT
Mil074.MQFLT      MQFLTCLLSLVVLTMVFNNVDGKPDCDPTCNGLKGRPSCTRCCYCYPAIGRKRADGHHSRMKEPASERSLLKLMSLY 77
Mus088.MQFLT      MQFLTCLLSLVVLTMVFNNVDGKPDCDPSCNGLKGRPSCTRCCYCYPAIGRKRADGHHSRMKEPAGERSLLKLMSLY 77
MRLPT
Cpt123.MRLPT      MRLPTMHSLFLMLLLMCAFYNIDGDDPGQAARGVDNGKFMSSLQSEGKPARFFMLGRKRRELCESKSCPDECGGVCRCGADGYCDDF   87
Mus089.MRLPT      MRLPTMHSLFLMLLLMCAFYNIDGDEPGQAARGVDHGKFMSSLQSEGKPARFFMLGRKRRELCESKSCPDECGGVCRCGADGYCDDF   87
Mil075.MRLPT      IRLPTMHSLFLMLLLMCAFYNIDGDEPGQAARGVDNGKFISSLQSEGKPARFFMLGRERRELCESKSCPDECGGVCRCGADGYCDDF   87
MSGLR
Mus090.MSGLR      MSGLRVVLLAHLLLVYLVTAKRDGDGLTKAFKNKRAATVFGRKITKSVQQPRTCPTMCPGGNECCSNML
MWKTG
Mil076.MWKTG      MWKTGAVLAVLLLSAQVTLQTEKDDGAVPWFKNSPLKRLLPRTCTTCSCASGPSGCTGSCCGSSCTCSSFSRGCGCST--  78
Cpt124.MWKTG      MWKTGAVLAVLLLSAQVTLQTEKDDGAVSWFKNSPLKRLLPRACSTCSCTSGPSGCTGSCCSSSCTCGAFSQGCGCATS-  79
Mus091.MWKTG      MWKTGAVLAVLLLSAQVTLQTEKDDGAMPWFRNSPLKRLLPRACSTCSCTSGPSGCTGSCCNSSCTCGAFSQGCGCATSG  80
Con-ikot-ikot
Cpt017.Con-ikot-ikot    MNMLVTLSVSAVVIVAATLFGSTPLHEPDLRQTDSEIDNETKECCANRTYDCLKKYPNREHLFHTLCHYYAAHPCGRRPVLGCCNGYMYCMGLYVQEYGLEVTHYGCSRRPC----    112
Cpt013.Con-ikot-ikot    MNMLVTLSVSAVVIVAATLFGSTPLHEPDLRQTDSEIDNETKECCANRTYDCLKKYPNREHLFHTLCHYYAAHPCGRRPVLGCCNGYMYCMGLHVQQYGLEKTHYGCSREPC----    112
Cpt018.Con-ikot-ikot    MNMLVTLSVSAVVIVAATLFGSTPLHEPDLRQTDSEIDNETKECCANRTYDCLKKYPNREHLFHTLCHYYAAHPCGRRPVLGCCNGYMYCMGLHVQQYGLEKTHYGCSRGNCSSLS    116
Mus020.Con-ikot-ikot    MYMLVTLSVSVVVVVAATLFGSTPLQEPDLRQTDSEIDNETKECCTNRTYHCLKKYPNREHLFHTLCHYYAAHPCGRRAFLGCCNGYMYCMGLHVQEYGLEVTHYGCSRLPC----    112
Cpt024.Con-ikot-ikot    MYMLVTLSVSVVVVVAATLFGSTPLQEPDLRQTDSEIDSETKECCTNRTYYCLKKYPNREHLFHTLCHYYAAHPCGRRPVLGCCNGYMYCMGLHVQQYGLEKTHYGCSREPC----    112
Cpt026.Con-ikot-ikot    MYMLVTLSVSVVVVVAATLFGSTPLQEPDLRQTDSEIDSETKECCTNRTYYCLKKYPNREHLFHTLCHYYAAHPCGRRPVLGCCNGYMYCMGLYVQEYGLEVTHYGCSRRPC----    112

Cpt019.Con-ikot-ikot    MAMNMWMTISVCVGVVMAASIFGSTPLQEGLS--RKNQIPCKCYRWGFHECL----SDLGGPWKKYLYPCDELARGECGSPP--RCCPGYFRCYAYCPLGDL---KDCSSKCKIVPC-  106
Cpt014.Con-ikot-ikot    --MTMSMTLCMFLMVAMLATGFGSTYLQGEKR-TIEDEEYHECCRLAMRNKFVSCMDEGIKTMDECWDCAYEEGANECGREPTDGCCPDFLECFSSELFHKDKDHQYAWNVCKSVDC-  114
Mil017.Con-ikot-ikot    MTMNMSMTLSVFVMVVIAATVLGSSPLKGKK------RAKPECCKRKMRKCFGCCIGFT-DDKSWCFDHCHYISATTCGYRPS-SCCKTFLDKYSACLFHSSYKASQCWKRTKHVPC-  109
Mil018.Con-ikot-ikot    -TTIMSMTLSVFVMIAIAATVLGSTELRDHV---VYDCTFQECCKFELRLCMEHCFDVA--TLSFCWTPCTQIAIKNCGLSPSTTCCPLFVDCYSSCLHAIHH-SYDCWNACKFAPCY  111
Cpt028.Con-ikot-ikot    -TTIMSMTLGVFVMFAIAATVLGSTELRDHVGLHVYDCAFHECCKFEMRLCMEDCFDLA--TLSFCWASCTQTAIVNCGIPTSTTCCPQFAHRYASCLNAGHY-PYYCWDTCKFEPCY  114
Mus021.Con-ikot-ikot    -TTIMSMTLGVFVMFAIAATVLGSTEPREHVGLHVYDCAFHECCKFEMRLCMEDCFDLA--TLSFCWASCTQTAIVNCGLPTSTTCCPQFAHRYASCLHAGHY-PYHCWDLYKFEPCY  114

Mil021.Con-ikot-ikot  MAMNAGMTLSMVVMVVLATAVGGSTPIEGIKGDSPGSERGRRDGVKGCCIEFIACHQQCSAPREVCWNQCWQEAPAACKARVSVGYCIAFQACHHNCDKNVHDCEQVCEYEAKYKYFEFPAITCKGFYEGFDDCVINTTLPAKYHCFDSNKDVAC   155
Cpt022.Con-ikot-ikot  MAMNAGMTLSMVVMVVLATAVGGSTPIEGIKGDSPGSERGRRDDVRRCCIEFMACHQQCRAPRDECWNQCWQETPAACKARVSIVYCIAFQACHHNCDGFVHDCVQVCEYEVKYKRFEFRAITCKGFNKAFGDCMSNTELPAKYYCFHSNKDVAC   155
Cpt021.Con-ikot-ikot  MAMNAGMTLSMVVMVVLATAVGGSTPIEGIKGDSPGSERGRRDDVRRCCIEFMACHQQCRAPRDECWNQCWQETPAACKARASIGHCIAFQACHHNCDGFVHDCLQVCEYEAKYKHFEFRAITCKGFNKAFGDCMSNTELPAKYYCFHSNKDVAC   155
Cpt027.Con-ikot-ikot  MAMNAGMTLSMVVMVVLATAVGGSTPIEGIKGDSPGSERGRRDDVRRCCIEFMACHQQCRAPRDECWNQCWQETPAACKARASIGHCIAFQACHHNC----------------------------------------------------------   97

Mil022.Con-ikot-ikot    LAMTMGMKMMTMVVLAVMATTVVCSVQLPEDQGDRLQRREQLSAQCCAEI-----QRCYD 55
Mil024.Con-ikot-ikot    MAVNLWM-SLGMLVVVVMATTVRDSTLLDEGERDLLDPKDDGSDGCCFEV-----IECYE 54
Cpt025.Con-ikot-ikot    MAVNLWM-SLGMLVVVVMATTVRDSTLLDEGERDPLEPKDDGSDGCCFEV-----IECWE 54
Mus012.Con-ikot-ikot    MAVNLWM-SLGMLVVVVMATTVRDSTLLDEGERDPLDPKDDGSDGCCFEV-----IACWE 54
Mus019.Con-ikot-ikot    --MTMSM-TLCMFLMVAMLATGFGSTYLQGEKR---TIEDEEYHECCRLAMRIKFGSCMD 54
Mus018.Con-ikot-ikot    MAVNMWM-TMGVLVVVLMA----------------------------------------- 18
Cpt023.Con-ikot-ikot    --------------IPLWT----------EQGL---CCNGVNNPYCCPYG----VHECVT 29
Mil019.Con-ikot-ikot    MAVNMWM-TMSVLVVVLMA----------EQGL---CCNGVNNPSCCHWG----VHECVS 42
Cpt029.Con-ikot-ikot    MAMKVW---ISVFVVVVMATTVIGFAPLQEQER---NGDDRSSRKCCAVK----SFMCLR 50
Mus011.Con-ikot-ikot    MAMKVW---ISVFVVVVMATTVIGFAPLQEQER---NGDDRSSRKCCAVK----SFMCLR 50
Mil025.Con-ikot-ikot    ------------------------------------------------------LYQCLR 6
Mil023.Con-ikot-ikot    MPMSMWM-TISVFVVAVMATTVIGSTPSHVQER---GRRSEA-NKCCAMR----VYTCLK 51
Cpt015.Con-ikot-ikot    -AMNMWM-TISILVVVVMAATVVGSTPLQEQGL---SRRTQTMRECCLEK----THNCTT 51
Mus015.Con-ikot-ikot    MAVNMWM-TISVCVVIVMAATVVGSSPSQDQGL---SRNDRQHSDCCLWE----FYDCLH 52
Mus016.Con-ikot-ikot    MAVNMWM-TISVCVVIVMAATVVGSSPSQDQGL---SRNDRQHSDCCLWE----FYDCLH 52
Cpt020.Con-ikot-ikot    MAVNMWM-TISVCVVVVMAATVVGSPPSQDQGL---SRNDRKNSACCLWE----FYDCLY 52
Mil020.Con-ikot-ikot    MTVNMWM-TISVCVVVVMAATVVGSPPSQDQGL---SLNDRTNSVCCLHE----FSDCLY 52
Mus009.Con-ikot-ikot    ---------------------------LQEEGL---SRKNQIPCKCYRWA----FHDCLS 26
Mus010.Con-ikot-ikot    ---------------------------LQEEGL---SRKNQIPCECYRIR----FQECLS 26
Mus014.Con-ikot-ikot    MAVNMWM-TISVCVGVVMAASIFGSTPLQ-EGL---SRKNQIPCKCYRWG----FHECLS 51
Cpt016.Con-ikot-ikot    MAMNMWM-TISVCVVVVMAATIVGYTPLQDEGL---SRKKRIACECYRWA----FYDCLS 52
Mus013.Con-ikot-ikot    MAVNMWM-TISVCVGVVMAASIFGSTPLQ--GL---SRKNQIPCECYRWA----FYDCLS 50
Mus017.Con-ikot-ikot    MAVNMWM-TISVCVVVVMAATIVGSTPLQ-EGL---SRKNQIPCECYRWA----FYDCLS 51

Mil022.Con-ikot-ikot    VCVD------SGLVVECWDHCRIRKSRMCGEGPSAYYCSKYSDCCYSCEDF--------- 100
```

```
Mil024.Con-ikot-ikot    QCLK------DGQAPYCWPWCRDGGRAICRRKDSERYCKMFHACYGICESH--------- 99
Cpt025.Con-ikot-ikot    QCVA------DGKVPYCLPWCRDEGRAICREKDSSRYCTVFHGCYDYCESQ--------- 99
Mus012.Con-ikot-ikot    QCVA------DGIVPYCLPWCRDEGRAICREKDSTRYCTVFHGCYDYCESQ--------- 99
Mus019.Con-ikot-ikot    EGIKTMD--------ECWNNAYKEGAEECGRGPTDGCCPDFLECFSTELFHKD------- 99
Mus018.Con-ikot-ikot    -----------------------ETRIYCQCE-DPHCCLGFLNCYQECAEDDRD--RSEI 52
Cpt023.Con-ikot-ikot    ECS-------DSDLNKCIDDCDAELRIYCQCE-DPHCCLGFLNCYIRCAVDDGD------ 75
Mil019.Con-ikot-ikot    ECT-------DQDLNKCIDDCDAVTRVECKCE-DPHCCLGFLNCYKQCALDDGD------ 88
Cpt029.Con-ikot-ikot    EHGCFTPPS-----SSCAQQCITTDTSSCGSTVGDNCCYGYMSCLVVHQIS-----PGSQ 100
Mus011.Con-ikot-ikot    QHGCFTPPS-----SSCAQQCKTTDTSSCGSTVGDNCCYGYMSCLVVHQIS-----PGSQ 100
Mil025.Con-ikot-ikot    DGGCLDRGS------SCHIACI-------FPNYCDICCQPYMLCVYNCLKA------SAN 47
Mil023.Con-ikot-ikot    DNNCI------EAQAQCDGPCA-------VPDDCLDCCTQYMHCAMNCIKYYEIPAQPED 98
Cpt015.Con-ikot-ikot    QSFITGDEDTYPLLVNCYGIYAG---MYCLSDEDTGCCFGFFDCYHNCAF---------- 98
Mus015.Con-ikot-ikot    QVV----ENWRRSLLFCYGQAAS------IC---SGCCSGYYDCFRRCPH---------- 89
Mus016.Con-ikot-ikot    QVV----ENWRRSLLFCYGQAAS------IC---SGCCSGYYDCFRRCPH---------- 89
Cpt020.Con-ikot-ikot    RIG----RDWRRNLLYCHSNAAT------IC---RGCCSGYYDCFRQCRH---------- 89
Mil020.Con-ikot-ikot    RMG----GDWRGNLLFCDGKAAT------IC---PDCCSGYYSCFRQCRH---------- 89
Mus009.Con-ikot-ikot    RFG----EDWKDHDDTCAKRARG------ECDSPPHCCPGYFRCFDTCKY---------- 66
Mus010.Con-ikot-ikot    QHD----ADWKRHLLPCSDMARG------ECDSPLRCCPGYYRCVAACND---------- 66
Mus014.Con-ikot-ikot    DLG----GPWKKYLYPCDELARG------ECGSPPRCCPGYFRCYAYCPL---------- 91
Cpt016.Con-ikot-ikot    HHG----GDWKNHMPGCSNMALG------ECESPPYCCPGYYRCIATCKN---------- 92
Mus013.Con-ikot-ikot    YHG----GDWKNHLPGCSDMARG------ECDSPSYCCPGYYRCVATCSD---------- 90
Mus017.Con-ikot-ikot    YHG----GDWKNHLPGCSDMARG------ECDSPSYCCPGYYRCVATCSD---------- 91

Mil022.Con-ikot-ikot    ---EGHECQDECTRDAKLTCMDIGAVECCP 127
Mil024.Con-ikot-ikot    ----LDGCARYCQLEMKWQCVVVGEGCC-- 123
Cpt025.Con-ikot-ikot    ---LFDDCADYCKLETKWQCVMVGEGCC-- 124
Mus012.Con-ikot-ikot    ---LFHDCAEYCKFETKWRCVLAGEGCC-- 124
Mus019.Con-ikot-ikot    --KDQQYAWNVCKG-G--P----------- 113
Mus018.Con-ikot-ikot    DSVALSWCYGHCRI-V--RCSV-------- 71
Cpt023.Con-ikot-ikot    ---SLSWCYSKCDI-V--SCLV-------- 91
Mil019.Con-ikot-ikot    ---ELTWCYSKCNL-A--SCLV-------- 104
Cpt029.Con-ikot-ikot    PGDPLLNSYNHCRLIY--PC---------- 118
Mus011.Con-ikot-ikot    PGDPLLNSYNHCRLIY--PC---------- 118
Mil025.Con-ikot-ikot    GEDVMRACHTSCKD-T--SCE--------- 65
Mil023.Con-ikot-ikot    RGDPLRDCHNQCKN-S--C----------- 114
Cpt015.Con-ikot-ikot    DAWPIDYCYDKCKD-V--PC---------- 115
Mus015.Con-ikot-ikot    R--DADVCYHLCKN-A--PC---------- 104
Mus016.Con-ikot-ikot    R--DADVCYHLCKN---------------- 101
Cpt020.Con-ikot-ikot    R--DPAVCYYRCQN-A--RC---------- 104
Mil020.Con-ikot-ikot    R--AADDCYRLCKN-E--PC---------- 104
Mus009.Con-ikot-ikot    S--DLKICSSKCKY-E--PC---------- 81
Mus010.Con-ikot-ikot    G--DLDICYFNCNF-A--PC---------- 81
Mus014.Con-ikot-ikot    G--DLKDCSSKCKI-V--PC---------- 106
Cpt016.Con-ikot-ikot    L--DLEICTSDCKY-D--PC---------- 107
Mus013.Con-ikot-ikot    L--VLKTCTSYCKN-A--PC---------- 105
Mus017.Con-ikot-ikot    L--VLKTCTSYCKN-A--PC---------- 106
```

Conkunitzin

```
Cpt030.Conkunitzin      YERQRWTERPVGQYFMDDINGKKEFMYTKFLSQVKFLQLRSRSAHQTMTYHCLNSRAFGTRFVLYDGEEIDSVETTYRRF    80
Mus022.Conkunitzin      YERQRWTERPVGQYFMDDINGKKEFMYTKYLSQVKFLQLRSRSAHQTMTYHCLNSRAFGTRFVLYDGEEIDSVETTYRRF    80
Mil026.Conkunitzin      YERQRWTERPVGQYFMDDINGKKEFMYTKYLSQVKFLQLRSRSAHQTMTYHCLNSRAFGTRFVLYDGEEIDSVETTYRRF    80

Cpt033.Conkunitzin  MATFN--IVLAALFASVILVDFAVQARRCHPPCEYGERCVAANRRERRHRHNNVCVPVRCLFQARRGRCLNFDRRYHFNTLTMSCTRVRTGACYGRNNRFSSSENCEL----------------------------------------------------------- 106
Mil027.Conkunitzin  MEGHRFAAVLILTICTLIL----------------GAWCLPGRAT---DGCSRSVALKKCYLPREPGSCETLEEKYYFDKYYNTCKTFDYSGCEGNDNRFNTMKDCKRCCPV-DVCYQPAEPGPCRAAFRRHYFNATTSKCEKFTYGGCHGNGNNFRSKEECYTKCG 147
Cpt032.Conkunitzin  ---------IMDCR----------------------------------KAIMLKDFNAICQLEADVGPCSGTFPRWFYNSGMSKCQLFDYGGCRGNENRFDTEEECMELC--------------------------------------------------------- 67
Mil028.Conkunitzin  ---------IMDCR----------------------------------KAIMLKDFNAICQLEADVGPCSGTFPRWFYNSGMSKCQLFDYGGCRGNENRFDTEEECMELC--------------------------------------------------------- 67
Cpt031.Conkunitzin  MTCFRSAVYLLLLT----------------------------------AAVNIGATYERCQLEPDTGPCRAAIPQFYYDRIEEKCLEFTYGGCGGNDNRFQTQAECEEACAF--DCDSQKVVGPCRAAFRRWYYNQDTKMCELFIYGGCGGNKNNYQSFADCQLYCS 131
Cpt034.Conkunitzin  --------------------------------------------------------------PAQPGNCRAFMPRYHYDADQGQCVRFTYGGCGGNSNNFETEAACNDFCRAEDICRMPKVVGPCRAGITRYYYDTASAACRQFIYGGCQGNLNNFGSLEACQGKC- 104
```

Coninsulin

```
Mus049.Insulin          ------------------------------------------------------------           0
Mil055.Insulin          --------------MARRLGFLIVALGLLLHWSH-----------------ADYEHIC--           27
Cpt079.Insulin          -------------------------MGILPSVCQRIAMREVTPALLAVLCLLGQATGQTF           35
Cpt077.Insulin          ---------------MTSSYLLLVALGLLLYVCQTS---------------LGSEHSCDS           30
Cpt080.Insulin          RHGVLRMALTWPSSPPVLLTLLLSLL--ALQLCAVY---------------GSYEHTCTL           43
Cpt078.Insulin          ------------MATGLLSLLLLAMLGFLLHVHVAR---------------AGLEHTCTL           33
Mil056.Insulin          ------------MATGLLSLLLLAMLGFLLHVHVAR---------------AGLEHTCTL           33

Mus049.Insulin          ---------------MKLAAVFVVLCAMLSLAEPKLHRIELHRMESTRQTLSAVGNSIKA           45
Mil055.Insulin          -NTEEHPTSQYYECGIEWPEYINEVCGVSGAS----------------------------           58
Cpt079.Insulin          CGVDSRPH-PKGICGRMLLRAHRTLCFLLSADYPNIFSRSIHKRSLKNIDDFPL-QAYAE           93
Cpt077.Insulin          ---SSTPH-PSGACGSNLADTQELMCEVEEELHREANSAR--------------------           66
Cpt080.Insulin          ATRSRGAH-PSGICGRNLARIVSVLCTPRG--YVSNWFTKRS--APNN-PAETF-VD---           93
Cpt078.Insulin          ETRRQGPH-PQGICGSKLPNIIHTVCQVMGRGYAGGQRLSRKRTSMIDSDDMEA-EE---           88
Mil056.Insulin          ETRRQGPH-PQGICGSKLPNIIHTVCQVMGRGYAGGQRLSRK------------------           74
                                                :*
Mus049.Insulin          LAFKYGLTSFNKPLGSTPEPLTNYLD-AQYY------------GVIGLGTPEQ-----QF           87
Mil055.Insulin          ------------------------------------------------GGERKRAKF-SQ           69
Cpt079.Insulin          MDLQRG-HSQDQPTGTSPNTETMRKLFVSLFPRVQTLLPDVGNSVLTQGGPGSKGPGGDH           152
Cpt077.Insulin          ---------------KKRGRDSWRRHFLSMVKT---------------RAKRNEAPP---           93
Cpt080.Insulin          -----Q---------NLRGVLLNKREALSYLRHRE-----------------PRAAR---           119
Cpt078.Insulin          -----G---------SRGGFLMSKRRALSYLQRET-------NPLV-MAGYRRRGPQ---           123
Mil056.Insulin          ------------------------------------------------------------           74

Mus049.Insulin          RVVFDTGSSNLWVPSKKCSLLDIACLLHNKYDSTKSST---YV------           127
Mil055.Insulin          LKKLRRRAALLNARANTY--HGSFCEC--CVHQCTENELEDHC------           108
Cpt079.Insulin          LQAKRAGDDL--LPHVQK--RGMVCDC--CYNVCQPSTLAQYCP-----           190
Cpt077.Insulin          -------------LQRAG--RGIVCEC--CYNSCSYEEWVEYCPAVTES           125
Cpt080.Insulin          -------------GTFGS--QGITCEC--CFNQCTYYELLQYCN-----           146
Cpt078.Insulin          -------------KRHGE--QGITCEC--CYNYCSTAQVR---------           146
Mil056.Insulin          -------------------------------------------------           74
```

Conodipine

```
Cpt035.Conodipine      MKLLASILWAMAALGVTWLVAADSSVQQLCQKYSNGCSTPVRVRCEEYFRPACDRHDSCY 60
Mus024.Conodipine      MKLLTSILWAMATLGVTWLVAADFPAQQLCQKYSNGCSTPVRVRCEEYFRPACDRHDSCY 60
Mil029.Conodipine      MKELESALWILAALALPRIAAQDSDTATSCARNSNGCSTFSFTPCKEHFRPACDRHDTCY 60
Cpt036.Conodipine      MKVLESALWILTALALPRIAAQDSDTATTCARNSNGCSTASITPCREHFRPACDRHDTCY 60
Mus023.Conodipine      MKVLESALWMLIALALPRIAAQDSDTATSCAENSNGCSTFSFTPCRQHFRPACDRHDTCY 60

Cpt035.Conodipine      RCGAHFGISRAECDEEFLFHMVRMCDQLG-PLSFCPRGTKRESPSRRTSPFIGLKERLME 119
Mus024.Conodipine      RCGAHFGISRAECDEEFLFDMVWMCDQLG-PRSFCPRGTKRESPSRRTSPFIGLKERLME 119
Mil029.Conodipine      ECGAHFNFTRVDCDNAFYYDMKECCNHGTDDEGNCPENRKRREASSMSITTPHRQLRLLE 120
```

```
Cpt036.Conodipine      GCGAHFNFTQNDCDNAFLEDMIARCDHGTDDEGNCPENRKRREASSTSIPTPLRQLRLLK 120
Mus023.Conodipine      ECGAHFNFTKDDCDNAFLEDMTARCDHGTDDEGNCPANQKRREASSMSITTPLRQLRLLE 120

Cpt035.Conodipine      NAAQPESPRDQDTRLIQFPSILCRHYAALYYIGVAVWAGAHYTTNADPRICPELKPCMPR 179
Mus024.Conodipine      NAAQPESPRDQDTRLIQFPSILCRHYAALYYIAVAVWGGAHYTTNADPRICPELKPCMPL 179
Mil029.Conodipine      KLMPPNSLSDGDPLQHHQRVRNCTEWAETYFGAVQLLGWLSFKHGVDATLCSQFEACMPK 180
Cpt036.Conodipine      KLMPPNSLSDRDPRQRHRRFWNC-------------------------------------- 143
Mus023.Conodipine      KFMPPNSLSDRDPRQRHRRSWNCTVWAKKYFDAVQWFGWLSFEDEVNATLCSQFEACMPQ 180

Cpt035.Conodipine      H 180
Mus024.Conodipine      H 180
Mil029.Conodipine      V 181
Cpt036.Conodipine      - 143
Mus023.Conodipine      V 181
```

Conoporin

```
Cpt041.Conoporin        ----------MAHVTLELE---------SDRDCREVPAREDPALTTEEAIDA-------- 33
Cpt040.Conoporin        ------------------------------------------------------------ 0
Cpt039.Conoporin        MGVPFPALKTMVTVVLLLMGNASQVVLSSDSTRVKLAAY--EAVTKGISLDGVTLQDLVA 58
Mil033.Conoporin        MGVPFPALKTMVTVVLLLMGNASPVVLSSVNTKVKLAAY--EAVTNGISLDGVTLQDLVA 58

Cpt041.Conoporin        ------------------------------IDSAMPRSIPAGWTGDAFPINRRKTVT--- 60
Cpt040.Conoporin        ------------------------------------------------------------ 0
Cpt039.Conoporin        PDYTVTCAFEVENWTRFPLMLPRLRIENSGAVTTSPTIIPPGK-REAFAVQKPNSNEGVY 117
Mil033.Conoporin        PDYMVTCALVVENWTRFPLMLPRLRIANSGAVTTSPTIIPPGK-REAFAVQKPDSNEGVY 117

Cpt041.Conoporin        ----IVVSAGNG-TLIL------------------------------------------- 72
Cpt040.Conoporin        --------------------------YSNKMGVGLTSPGLVDVVPGDDWFDQMYSGSNSD 34
Cpt039.Conoporin        GTISWEIEGARRRVVIMWSAPVNFGQHTNWMGLGMTREGILNEPSDKSWFSQMYNDQSSA 177
Mil033.Conoporin        GTISWEIEGAKQRVVIMWSAPFDFSQYSNWMGLGMTREGLVDAPSDRTWFDQMYYHSSNA 177

Cpt041.Conoporin        ------------------------------------------------------------ 72
Cpt040.Conoporin        DLTCKSKEFYYDMVPIVYRNGGV------------------------------------- 57
Cpt039.Conoporin        NLKFVRLEYDTNTDPFIYSDDRFEVEGVMTNGHKAQVKVVVRPITNNMEDLAQVIRQRLD 237
Mil033.Conoporin        ELTFNRMEYFFNTDPFTYIDSRFEVEGVMTNSHKVEMKIVLRPISHNLDDLAQVIRQRLE 237
```

Conopressin/Conophysin

```
Mil035.Conopressin/Conophysin       MKCSVLQMSRLSWAMCLMLLMVLLLGTAHGCFIRNCPRGGKRAVDAVQPTRQCMPCGPDG 60
Cpt042.Conopressin/Conophysin       MKCSVLQMSRLSWATCLMLLMLLLLGTAQGCFIRNCPRGGKRAVDAVQPTRQCMPCGPDG 60
Mus025.Conopressin/Conophysin       MKCSVLQMSRLSWATCLMLLMLLLLGTAQGCFIRNCPRGGKRAVDAVQPTRQCMPCGPDG 60
Cpt043.Conopressin/Conophysin       -------------------LLLLLVLTAQAYFVRNPSPDDKRDVQERDP------CMDCQ 35
Mil034.Conopressin/Conophysin       ----AMPMGR--PTPCL--LLLLLVLTAQAYFVRTPSPSDKRDVDERAA------CMYCQ 46

Mil035.Conopressin/Conophysin       MGQCVGPSVCCGLGLGCLMGTPETEVCQKENESAVPCAISGRHCGMDN----TGNCVAD- 115
Cpt042.Conopressin/Conophysin       MGQCVGPSVCCGLGLGCLMGTPETEVCQKENESAVPCAISGRHCGMDN----TGNCVAD- 115
Mus025.Conopressin/Conophysin       MG---------------------------------------------------------- 62
Cpt043.Conopressin/Conophysin       FGQCVGPKTCCGT-KGCEMGTVLASVCLEE----DPCQIFGWPCSMNGDTIKNGYCVGGG 90
Mil034.Conopressin/Conophysin       FGQCVGPKICCGD-KGCEMGSKLANVCVLEEENGKPCQVFGWPCSMNGDTTENGRCVGGG 105

Mil035.Conopressin/Conophysin       -GICCVEDACSFNSLC------------------------------  130
Cpt042.Conopressin/Conophysin       -GICCVEDACSFNSLCRVDTDQEDSVSARQELLTLIRRLLVNRQYD  160
Mus025.Conopressin/Conophysin       ----------------------------------------------  62
Cpt043.Conopressin/Conophysin       IGVCCTFGSCVINPYCD-----------------------------  107
Mil034.Conopressin/Conophysin       IDICCAFDTCVVNLKCKK----------------------------  123
```
