# Supplementary material for: Diversity and Novelty of Venom Peptides in Vermivorous Cone Snails, Subgenus Rhizoconus (Gastropoda: Mollusca)
Source: Mar Drugs. 2025 Jun 26;23(7):266. doi: 10.3390/md23070266 (PMC12300008; doi:10.3390/md23070266)

Supplemental File S5. Conopeptide abundance vs. expression levels of top 10 most highly expressed gene superfamilies per species.

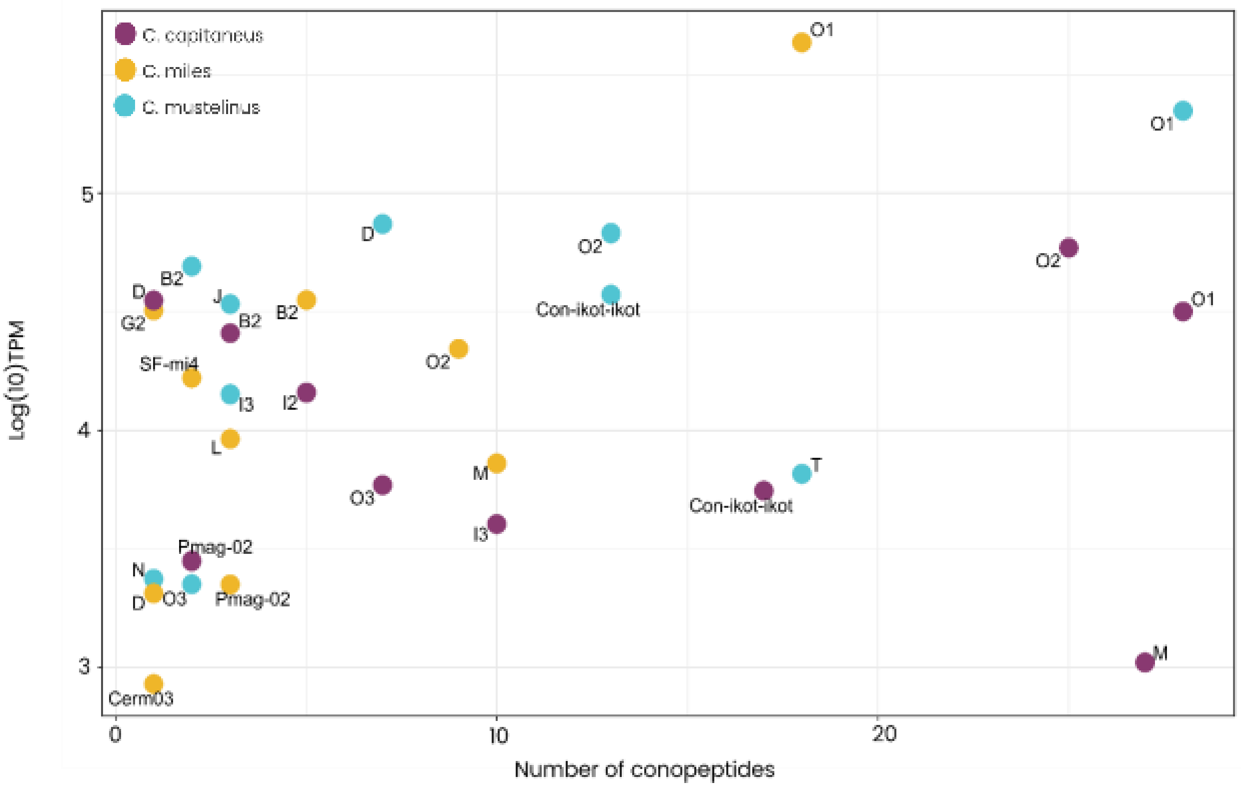

Supplement: Supplementary file 1 [file marinedrugs-23-00266-s001.zip › Supplementary Files/Supplementary File S5. Abundance and expression levels - Copy.pdf]
